# Decoding the endometrial niche of Asherman’s Syndrome at single-cell resolution

**DOI:** 10.1038/s41467-023-41656-1

**Published:** 2023-09-21

**Authors:** Xavier Santamaria, Beatriz Roson, Raul Perez-Moraga, Nandakumar Venkatesan, Maria Pardo-Figuerez, Javier Gonzalez-Fernandez, Jaime Llera-Oyola, Estefania Fernández, Inmaculada Moreno, Andres Salumets, Hugo Vankelecom, Felipe Vilella, Carlos Simon

**Affiliations:** 1 Carlos Simon Foundation, INCLIVA Health Research Institute, Valencia, Spain; 2 Department Ob/Gyn Vall d’Hebron Institut de Recerca, Barcelona, Spain; 3 Igenomix R&D, Valencia, Spain; 4 Department of Pediatrics Obstetrics & Gynecology, University of Valencia, Valencia, Spain; 5 Department of Obstetrics and Gynaecology, Institute of Clinical Medicine, University of Tartu, Tartu, Estonia; 6 Competence Centre on Health Technologies, Tartu, Estonia; 7 Division of Obstetrics and Gynaecology, Department of Clinical Science, Intervention and Technology (CLINTEC), Karolinska Institute and Karolinska University Hospital, Stockholm, Sweden; 8 Department of Development and Regeneration, Cluster of Stem Cell and Developmental Biology, Unit of Stem Cell Research, University of Leuven (KU Leuven), Leuven, Belgium; 9 Department of Obstetrics and Gynecology, Beth Israel Deaconess Medical Center, Harvard Medical School, Boston, MA USA

**Keywords:** Infertility, Preclinical research, Translational research, Molecular medicine

## Abstract

Asherman’s Syndrome is characterized by intrauterine adhesions or scarring, which cause infertility, menstrual abnormalities, and recurrent pregnancy loss. The pathophysiology of this syndrome remains unknown, with treatment restricted to recurrent surgical removal of intrauterine scarring, which has limited success. Here, we decode the Asherman’s Syndrome endometrial cell niche by analyzing data from over 200,000 cells with single-cell RNA-sequencing in patients with this condition and through in vitro analyses of Asherman’s Syndrome patient-derived endometrial organoids. Our endometrial atlas highlights the loss of the endometrial epithelium, alterations to epithelial differentiation signaling pathways such as Wnt and Notch, and the appearance of characteristic epithelium expressing secretory leukocyte protease inhibitor during the window of implantation. We describe syndrome-associated alterations in cell-to-cell communication and gene expression profiles that support a dysfunctional pro-fibrotic, pro-inflammatory, and anti-angiogenic environment.

## Abstract (web summary)

Asherman’s Syndrome is characterized by intrauterine scarring and infertility. Here, the authors decode the Asherman’s Syndrome endometrial cell niche by single-cell RNA sequencing and in vitro analyses of patient-derived endometrial organoids.

## Introduction

Asherman’s Syndrome (AS) is an acquired pathological condition defined by the presence of intrauterine adhesions (IUAs) that cause the uterine walls to adhere to each other, which results in menstrual abnormalities, pelvic pain, infertility, recurrent miscarriage, and abnormal placentation^1^. Affecting 4 in every 10,000 women^2^, AS is considered a rare disease (Orphanet database; ORPHA:137686). Trauma or infection induced by procedures such as curettage or cesarean section (mainly in the gravid uterus) can disrupt the endometrial basalis and prompt the onset of AS^3–7^.

Since the first report of AS as traumatic amenorrhea more than a century ago^8^, treatment strategies have focused on the recurrent surgical removal of IUAs. Preventative measures have had limited clinical results, especially in the moderate and severe stages refractory to surgical treatment^9,10^. AS arises due to the iatrogenic disruption of the endometrial niche^11^ and while bulk tissue-based transcriptomics studies^12–14^ have provided a degree of insight, convincing evidence regarding the detailed molecular mechanisms involved in endometrial dysfunction remains elusive. Advancements in single-cell RNA sequencing (scRNA-seq)-based analysis of the human endometrium^15^ and endometrial organoid culture systems^16^ have enabled a deeper understanding of the cellular cartography and cell-to-cell communication (CCC) in endometrial pathologies such as endometriosis^17^ and atrophic endometrium^18^.

Deciphering the cellular complexity and heterogeneity of the endometrial cell niche will provide a means to understand the etiological causes of IUA formation and endometrial dysfunction in AS. Therefore, we performed systematic differential cellular, transcriptomic, and immunological comparisons at single-cell resolution and explored differential CCC within the normal and AS endometrium in vivo. Furthermore, we evaluated AS patient-derived endometrial epithelial organoids (EEOs) in vitro at the single-cell level to support the functional relevance of our findings.

Our data highlight significant losses in the epithelial compartment and differentiation through Wnt and Notch pathway disruption in AS, which significantly impacts functionality during the window of implantation (WOI). We describe a characteristic secretory leukocyte protease inhibitor (SLPI)-expressing stressed epithelium, and alterations in gene expression profiles and CCC, which induce the dysfunctional pro-fibrotic, pro-inflammatory, and anti-angiogenic environment associated with AS. These findings provide a platform for a deeper understanding of AS, which may support the development of improved preventative and therapeutic approaches.

## Results

### Single-cell analysis reveals alterations in the AS endometrium

**Fig. 1:**
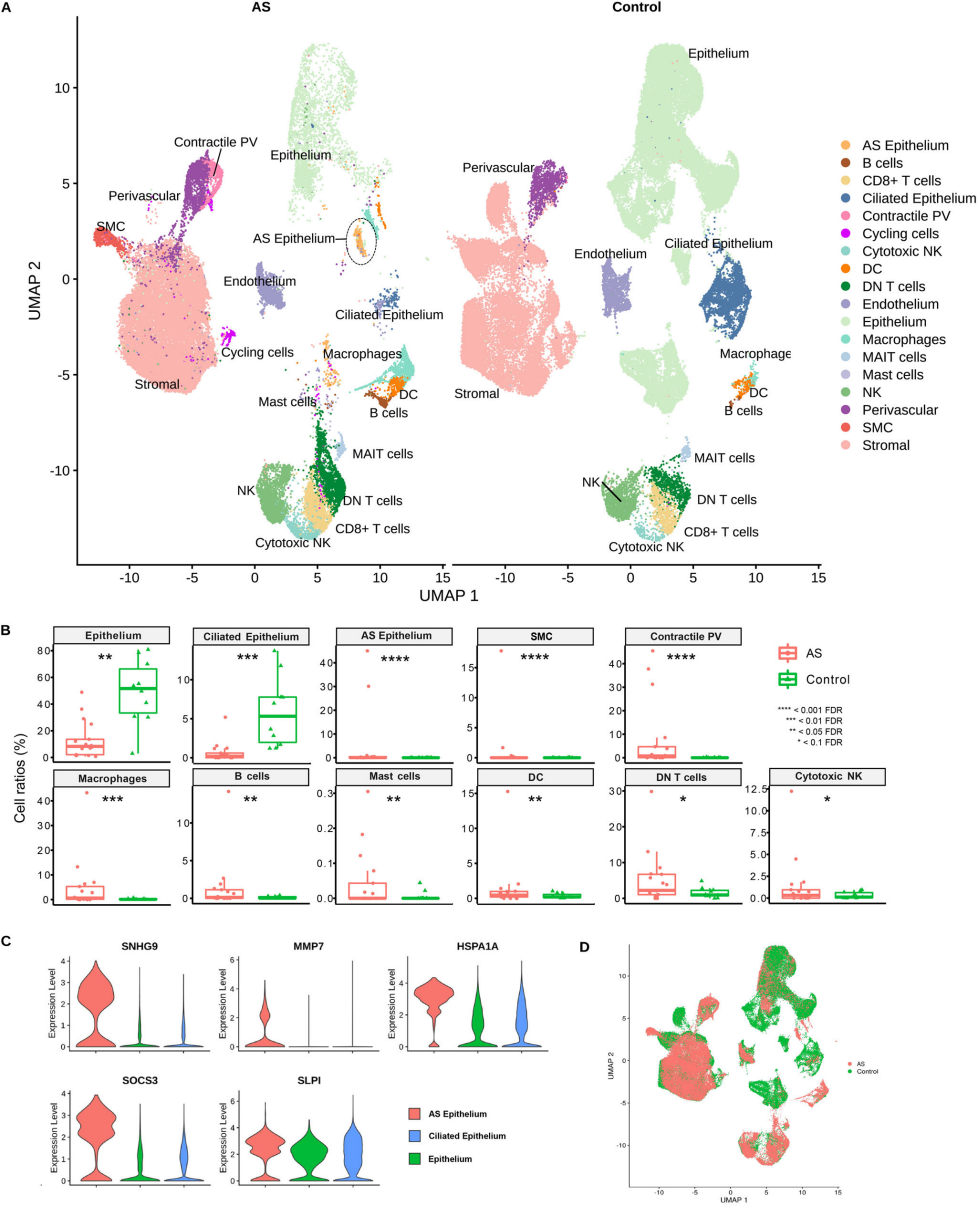
Single-cell RNA-sequencing cartography of the human endometrium in Asherman´s syndrome patients and healthy controls. **A** UMAP integration of 109,538 cells from nine AS patients (40,336 cells) and ten healthy individuals (Control) covering the secretory phase from the GSE111976 dataset (69,202 cells). Characteristic AS epithelium highlighted within a dotted circle. **B** Cell ratios comparing the endometrium of healthy (Control) and AS patients (two-sided NB-GLM statistical test; *n* = 9 for independent AS patients, *n* = 10 for independent control endometrium). **C** Marker gene expression in AS epithelium. **D** Integrated UMAP highlighting characteristic AS types. AS Asherman’s syndrome, DC dendritic cells, DN double negative, FDR false discovery rate, MAIT mucosal-associated invariant T, NK natural killer, PV perivascular, SMC smooth muscle cells.

To explore whether a possible displacement of the WOI may also be present in AS patients as another plausible dysfunctional cause of infertility even in an HRT medicated cycle, scRNA-seq was initially applied to characterize the differential endometrial cartography of AS through a comparison of 175,602 cell transcriptomes, including 69,202 control transcriptomes covering the secretory phase^15^ (secretory controls), and 66,064 WOI controls (Fig. 1; “Methods”; Supplementary Figs. 1 and 2; and Supplementary Data 1). We generated a comparative cellular landscape of the dysfunctional AS endometrium by exploration of cluster marker genes (Supplementary Data 2, 3, and 4), which we compared to annotated major cell types in reported endometrial atlases^15,19^.

We identified eighteen cell types in our AS endometrial atlas (Fig. 1A; Supplementary Fig. 3). Epithelial cells (including the ciliated subpopulation), stromal fibroblasts, perivascular (PV) cells, endothelial cells, immune populations (macrophages, dendritic cells, natural killer (NK) cells, cytotoxic NK cells, CD8+ T cells, double-negative (DN) T cells, and a specific population of innate-like T cells defined by their semi-invariant αβ T cell receptor (TCR), denominated as mucosal-associated invariant T (MAIT) cells^20^.

Epithelial cells specifically expressed metallothionein (MT) family genes (*MT1G, MT1H, MT1X*, and *MT1M*), which are associated with the early secretory phase (Supplementary Fig. 3A; Supplementary Data 2, 3, and 4). Interestingly, a characteristic cell type in AS was identified beyond the expected endometrial cell populations (Fig. 1A, B) - a stressed epithelial subpopulation expressing elevated levels of SLPI and co-expressing cell stress-related genes (i.e., *HSPA1A* and *SOCS3*) - that we define as “AS epithelium” (Fig. 1C, D). We defined two PV populations: pericyte/mural cells expressing regulator of G protein signaling 5 (*RGS5*) and contractile PVs expressing genes involved in contractile functions (e.g., *ACTA2, TAGLN*, and *MYH11*) (Fig. 1A; Supplementary Fig. 3A, B; Supplementary Fig. 4A, B). The immune cell population included a myeloid lineage cluster, which expressed classic macrophage markers (e.g., *LYZ*) and genes encoding S100 calcium-binding proteins (*S100A8* and *S100A9*), and dendritic cells, which expressed regulatory antigen processing CD74 and HLA class II genes. We discovered several lymphoid lineage cell clusters, including NK cells, expressing *NKG*7. We identified cytotoxic NK cells by the expression of serine protease genes (*GZMH* and *GZMB*) and cytolytic-related gene *PRF1* and CD8+ T cells by the expression of *IFNG, IL32*, and *CCL5*. We identified a population of double-negative (DN) T cells through *IL7R, LTB, CD52*, and *CD3D* expression, and the MAIT^20^ cell population through a specific expression profile of *IL23R, IL4I1, LTB, IL7R*, and *KLRB1* (Supplementary Fig. 3B), which denotes cells that have an innate capacity to respond to a particular ligand set without memory T cell expansion and development (Fig. 1A).

We next discovered significant differences in cell population ratios between AS and secretory controls (Fig. 1A, B). Importantly, we confirmed a significant reduction in the epithelium (8.3% vs. 51.65%) and ciliated epithelium (0.19% vs. 5.3%) in the AS endometrium. Myeloid and lymphoid cell lineages became more abundant, with a highly significant increase in macrophages (0.6% vs. 0.08%), B cells (0.13% vs. 0.015%), dendritic cells (0.45% vs. 0.18%), DN T cells (2.22% vs. 0.94%), and cytotoxic NK cells (0.32% vs. 0.11%). We also observed an augmented number of cells in the contractile PV population (0.74% vs. 0.01%) (Fig. 1B).

We also encountered differential gene expression profiles in several cell compartments when comparing AS to secretory control endometria (Supplementary Fig. 5, Supplementary Data 5). Secretory activity-related gene expression in the epithelium (MT family genes and secretogoblin genes – *SCGB1D2* and *SCGB2A*1) became downregulated in AS. We observed the upregulated expression of extracellular matrix (ECM) fiber genes (collagens and fibronectin *(FN1*)) in the stroma, and anti-angiogenic genes *(IGFBP5*^21^ and *IGFBP6*^22^) in the endothelium. In general, the immune cell population overexpressed genes related to pro-inflammatory processes in AS. Macrophages exhibited upregulation of S100 family genes (*S100A8/9/12*)^23^ and the long non-coding RNA NEAT1 associated with inflammation and immune activation^24^. CD8+ T cells, DN T cells, and NK cells overexpressed cytotoxic granulysin (*GNLY*) in AS, while MAIT cells possessed a cell stress-related transcriptomic profile (Supplementary Fig. 5).

By establishing a single-cell RNA-seq atlas of AS endometria, we demonstrate the ability to track changes in cellular composition and transcriptomic heterogeneity. These data help to describe the previously unknown tissue context of AS in the absence of IUAs.

### AS cartography in the context of the WOI in moderate to severe disease

**Fig. 2:**
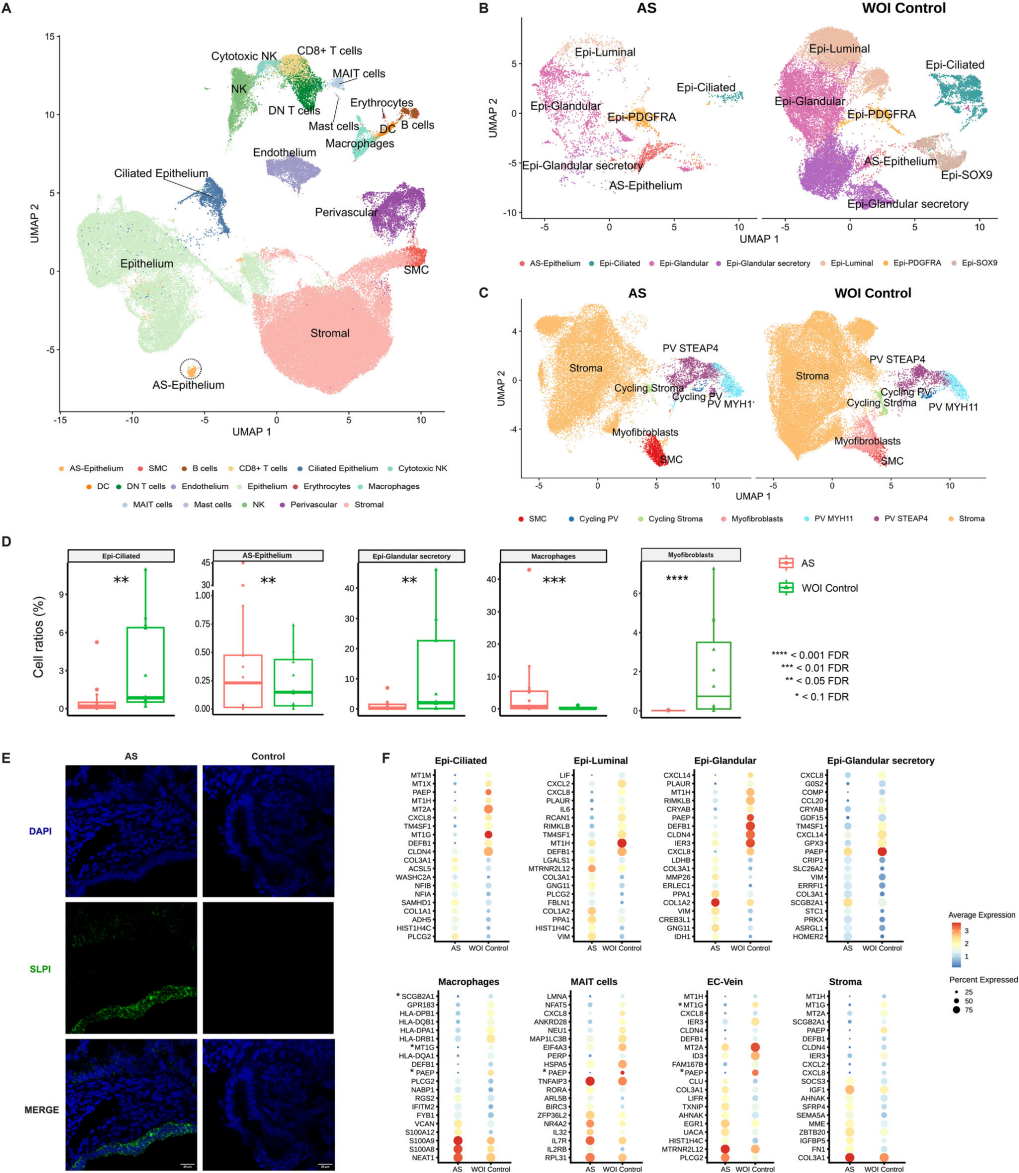
Single-cell RNA-sequencing-based fine-grain identification of epithelial and stromal subpopulations in Asherman’s syndrome patients compared to healthy window of implantation controls. **A** UMAP integration of scRNA-seq data obtained from 106,400 cells, including nine AS patients (*n* = 40,336 cells) and twelve healthy WOI controls (*n* = 66,064 cells) showing the main cell types present in the endometrium during the WOI. Specific zoom-in UMAP of **B** epithelial cell composition and **C** stromal and PV cell compositions. **D** Ratios of specific cell types comparing AS patients to WOI controls (two-sided NB-GLM statistical test; *n* = 9 for independent AS patients, *n* = 12 for independent WOI control samples). **E** Representative RNAscope images showing the expression of SLPI as a marker of the characteristic AS epithelium cell type in AS patients. Images show an example of SLPI expression in AS and control endometria. SLPI (green) expression counterstained with DAPI shown merged and in separate channels for clarity. Scale bar = 20 µm. **F** Differential gene expression dot plots for epithelial subtypes, macrophages, MAIT cells, EC-Vein, and stroma in AS and WOI control endometria (two-sided Wilcoxon Rank Sum test; FDR < 0.05). Note: the genes marked with and asterisk (*) in the differential expression results may have been influenced by ambient RNA ambient contamination. AS Asherman’s syndrome, DC dendritic cells, DN double negative, Epi epithelial, FDR false discovery rate, MAIT mucosal-associated invariant T, NK natural killer, PV perivascular, SLPI Secretory Leukocyte Peptidase Inhibitor, SMC smooth muscle cells, WOI window of implantation.

To resolve endometrial dysfunction associated with the WOI at a finer level of detail, we compared WOI AS vs. WOI controls by a refined cell type annotation (Fig. 2A–C) using supportive canonical marker expression (Supplementary Fig. 6A). We discovered a considerable reduction in glandular secretory and ciliated epithelial subpopulations together with an increase in the distinctive SLPI + AS epithelium and immune populations (Fig. 2A–D; Supplementary Fig. 6B). RNAscope experiments localized SLPI+ cells to the luminal and glandular epithelium of AS patients (Fig. 2E). We also observed an epithelial cell population we refer to as “epithelial-PDGFRA” that expressed a mixed combination of epithelial and stromal markers.

Differential gene expression analysis identified a significant downregulation of endometrial receptivity-associated genes in epithelial subtypes in AS, specifically *PAEP, GPX3*, and *CXCL8* expression in glandular and ciliated subtypes and *IL6* in the luminal epithelium (Fig. 2F; Supplementary Data 6).

Given the dependence of the endometrial activity on estradiol and progesterone, we interrogated the comparative expression of *ESR1* and *PGR* during the WOI. We observed *ESR1* and *PGR* overexpression in the glandular, glandular secretory, luminal epithelial subpopulations, and stromal compartments of AS compared to control endometria (Supplementary Fig. 6C, D). These findings agree with published studies^25,26^ that reported overexpression of *ESR1* in the endometrium of patients with IUAs. As for *PGR*, prior studies were conducted in the proliferative phase^26,27^, whereas our comparison focused on the WOI.

**Fig. 3:**
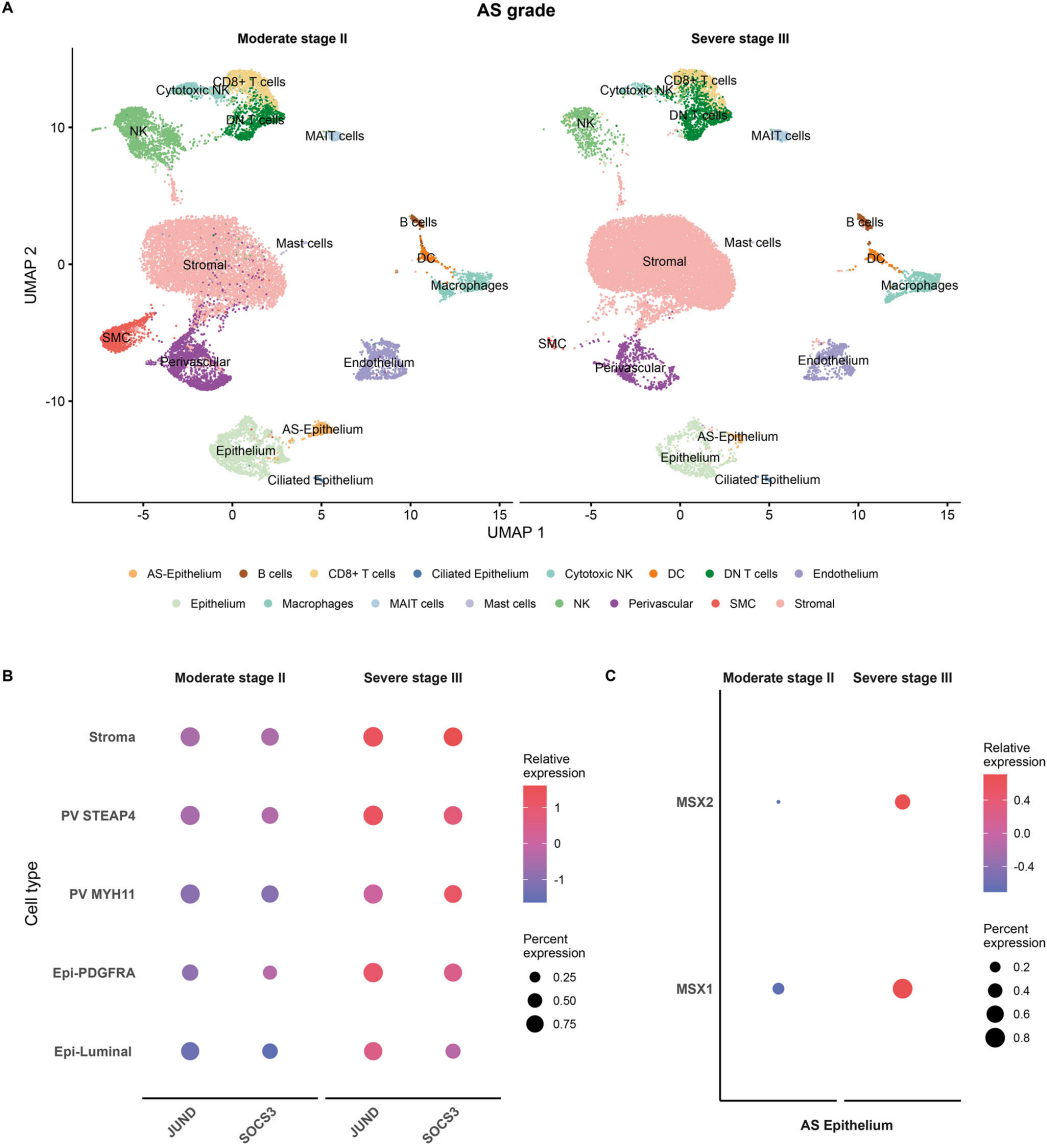
Single-cell RNA-sequencing-defined alterations according to Asherman’s syndrome severity. **A** UMAP displaying the primary cell population detected in AS patients with moderate (Stage II) and severe (Stage III) forms of the disease. **B** Dot plot of differential gene expression analysis between AS severity groups (two-sided Wilcoxon Rank Sum test; FDR < 0.05). **C** Dot plot of the differential expression analysis of MSX genes in AS epithelium stratified by AS severity. AS Asherman’s syndrome, Epi epithelium, PV perivascular, UMAP uniform manifold approximation and projection.

To explore differences between moderate and severe AS and further understand disease development, we compared single-cell data from four patients with severe AS (#05, 08, 10, and 13) to five patients with moderate AS (#07, 09, 12, 14, and 15). (Fig. 3A; Supplementary Data 1). We annotated cell types following AS cartography at finer resolution in the WOI (Supplementary Fig. 7A, B) and first observed a general reduction in all epithelial cell subtypes in severe AS (Fig. 3A; Supplementary Fig. 7A). Differential gene expression analysis by cell type also revealed a significant upregulation of stress response genes (*JUND* and *SOCS3*) in the stroma, PV subpopulation, PDGFRA epithelium, and luminal epithelium (Fig. 3B) and of *MSX1* and *MSX2* expression in the AS epithelium in severe compared to moderate AS (Fig. 3C).

These data describe a dysfunctional AS epithelium characterized by a loss of the glandular secretory cellular compartment and its transcriptomic receptivity program and the presence of a stressed SLPI + AS epithelium. The increased number of immune cells (particularly pro-inflammatory macrophages) defined the altered AS microenvironment. We also reveal that a stress response gene expression profile characterizes the advance from the moderate to severe form of AS.

### Aberrant cell-to-cell communication underlies the dysfunctional cellular environment of the AS endometrium

**Fig. 4:**
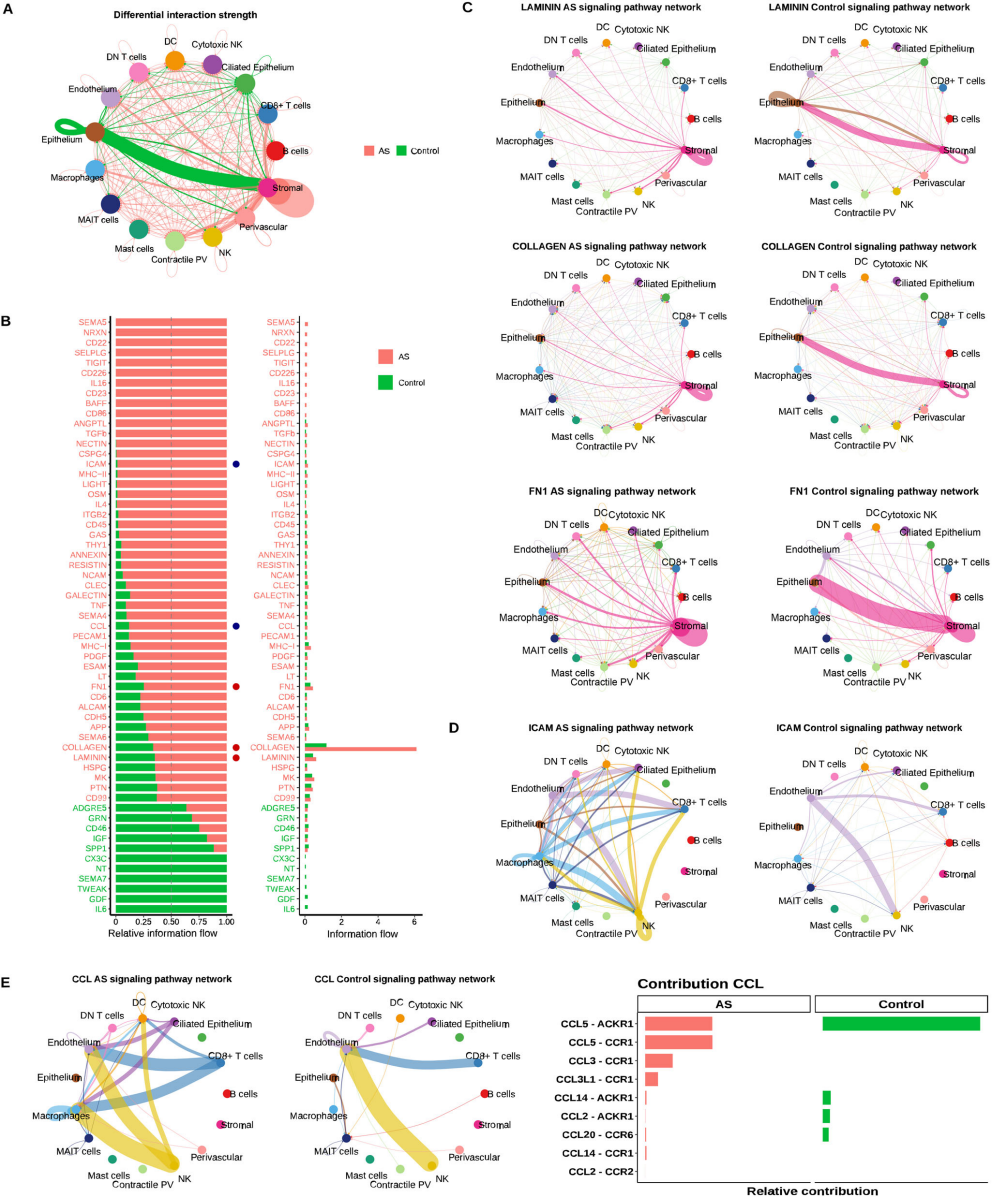
Differential cell-to-cell communications between Asherman’s syndrome and control endometria. **A** Chord plot displaying differentially active CCC comparing AS and control endometria. Arrows between cell types depict the direction of interactions. Relative thicknesses of red (AS) and green (Control) arrow lines represent the expression-based strength of the interaction between cell types. **B** Relative and absolute flows of differentially active signaling pathways between AS and control endometria (two-sided Wilcoxon test; FDR > 0.05). Blue dots = pathways involved in immune cell recruitment and inflammation. Red dots = pathways involved in ECM and fiber formation. CCC chord plots of **C** LAMININ, COLLAGEN, FN1, and **D** ICAM signaling pathways in AS and control endometria. Arrows follow the color code of cell types commonly detected in AS and control cartographies. **E** CCC chord diagrams of CCL signaling pathway in AS and control endometria (left panel). Ligands and receptors contributing to CCCs of the C-C motif chemokine ligand (CCL) signaling pathway (right panel). AS Asherman’s syndrome, DC dendritic cells, DN double negative, MAIT mucosal-associated invariant T, NK natural killer, PV perivascular, SMC smooth muscle cells.

CCC plays a critical role in maintaining tissue homeostasis; to dissect the complex interactions between endometrial cell types and their possible interruption in AS, we inferred intercellular CCCs by analyzing the expression of ligand-receptor pairs using CellChat^28^. We calculated communication information flows – the total interaction probability among all pairs of cell populations present in the AS endometrial atlas, and analyzed differential interaction probabilities between AS and secretory controls for each pathway (Fig. 4; Supplementary Fig. 8).

Consistent with the pro-fibrotic nature of AS, the CCC network analysis demonstrated a significant loss of normal interactions between epithelial and stromal cells, which shifted to stromal self-stimulation (Fig. 4A) through the production of ECM components (collagen, *FN1*, and laminin) (Fig. 4B, C; Supplementary Figs. 8–11).

Immune cells displayed increased signaling interactions with the endothelium through ICAM and CCL in AS (Fig. 4D, E; Supplementary Fig. 12), which suggests both pro-inflammatory and immune cell recruitment activities of the blood vessels^29^. In addition, we observed an enrichment of signaling pathways related to major immune processes (e.g., *ICAM, MHC-II and MHC-I, LIGHT, IL4, RESISTIN, NCAM*, and *CCL*) (Fig. 4B; Supplementary Fig. 8B).

**Fig. 5:**
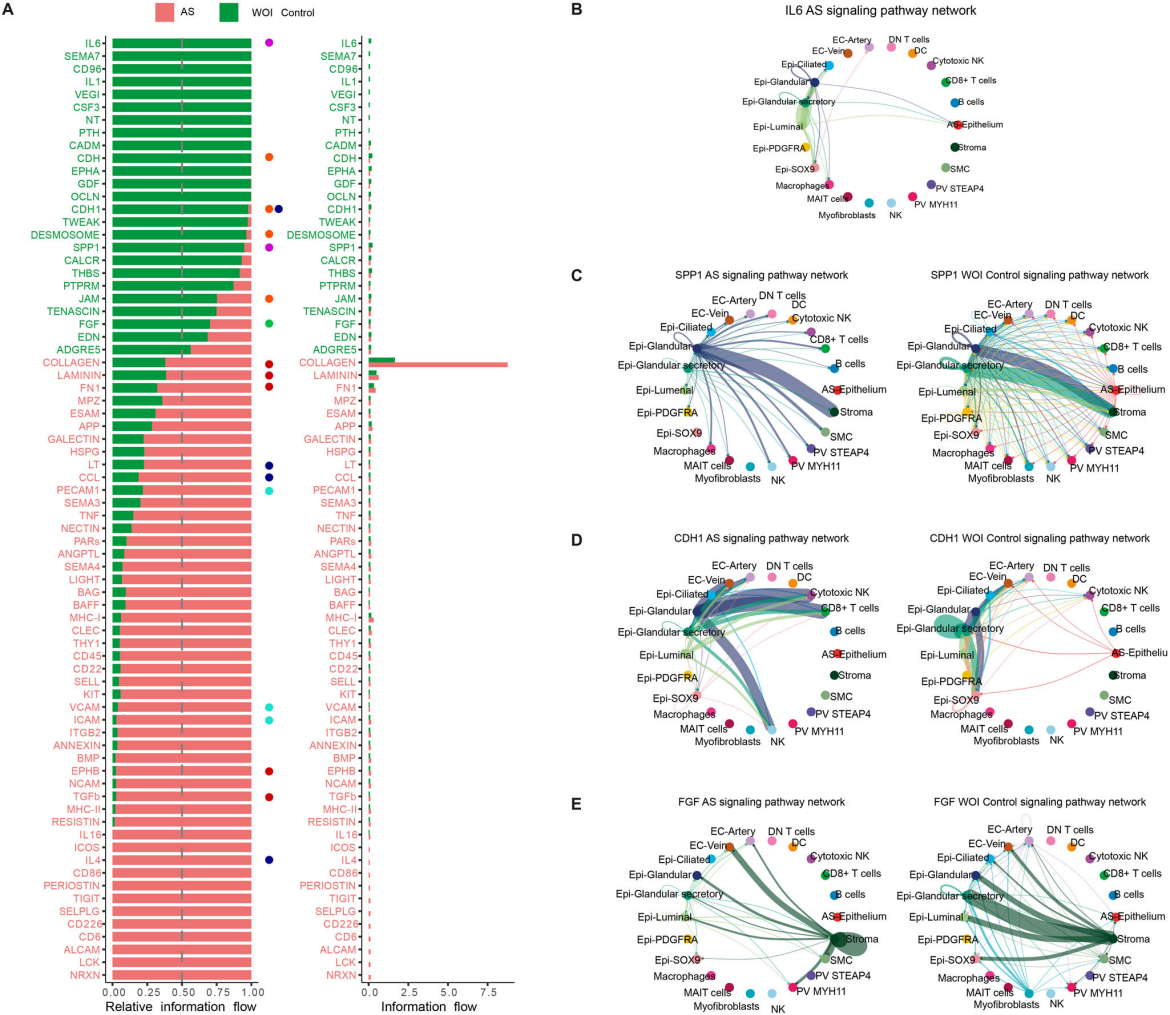
Molecular profiling and refined cell-to-cell communication analysis of Asherman’s syndrome compared to control endometria during the window of implantation. **A** Relative and absolute information flow for each differentially detected signaling pathway differentially detected in AS and WOI control endometria (two-sided Wilcoxon test, FDR > 0.05). Blue dots = immune system signaling pathways. Red dots = ECM- related pathways. Green dots = pathways involved in epithelial development. Cyan dots = pathways related to immune cell recruitment. Purple dots = signaling pathways of the WOI and decidualization processes. Orange dots = signaling pathways related to cell junctions and cellular unions. CCC chord diagrams displaying **B** IL6 (AS only), **C** SSP1, **D** CDH1, and **E** FGF signaling pathways in AS and WOI control endometria. AS Asherman’s syndrome, DC dendritic cells, DN double negative, Epi epithelial, FDR false discovery rate, MAIT mucosal-associated invariant T, NK natural killer, PV perivascular, SMC smooth muscle cells, WOI window of implantation.

A subsequent differential CCC analysis in the WOI context (Fig. 5A) revealed the decreased communication probability of canonical and non-canonical (nc)WNT pathways due to the specific absence of WNT7A-FZD6/LRP6 interactions in SOX9-epithelial cells and WNT5A-FZD6 interaction between stromal and luminal cell subtypes in AS (Supplementary Fig. 13A, B), which may slow luminal cell differentiation (Supplementary Data 7 provides a comprehensive view of CCC results). NOTCH pathway signaling significantly decreased in glandular, glandular secretory, and luminal subtypes, specifically in the JAG1-NOTCH2 ligand-receptor pair, which correlates with the dysregulated differentiation of glandular epithelium subtypes in AS (Supplementary Fig. 13A, B).

The WOI-focused analysis identified a loss of connectivity between all epithelial subtypes involving IL6 (Fig. 5B), a well-known epithelial hallmark for pregnancy establishment/maintenance^30^, and dysregulation of SPP*1* (Osteopontin) and *CDH1* (Fig. 5C–E; Supplementary Fig. 14A) in AS. SPP1, an ECM-related protein involved in cell attachment, is crucial for stromal decidualization^31^ while the CDH1 cadherin mediates cell-cell epithelial adhesions. We detected impaired DESMOSOME and JAM signaling between luminal, ciliated, glandular, and glandular secretory epithelial subtypes in AS (Fig. 5D; Supplementary Fig. 14A, B), which suggests the loss of epithelial structural integrity. Interestingly, we observed increased CDH1-KLRG1-mediated communication of the epithelium with NK and CD8+ T cell subpopulations in AS, which creates a pro-inflammatory microenvironment adjacent to the luminal epithelium^32^. We identified several over-represented pathways facilitating communication between immune and glandular epithelial cells in AS (e.g., *LCK, ALCAM, CD6, CD226, SELPLG, TIGIT, CD86, IL4*, and *ICOS*). We also detected altered fibroblast growth factor (FGF) signaling between the epithelium and stroma - reduced *FGF7-FGFR1* signaling accompanied by activated *FGF2-FGFR1* signaling (Fig. 5A, E; Supplementary Fig. 14C).

Our results reveal a marked dysfunction in cell-to-cell and cell-to-matrix interactions along the stromal-epithelial axis in AS, which leads to a pro-fibrotic environment and disrupted epithelial differentiation with uncoupled *WNT/NOTCH* signaling pathways during the WOI. In parallel, immune CCC becomes activated, and blood vessels increase leukocyte/myeloid recruitment through a chemokine-rich pro-inflammatory microenvironment in AS.

### AS patient-derived endometrial epithelial organoids recapitulate AS

**Fig. 6:**
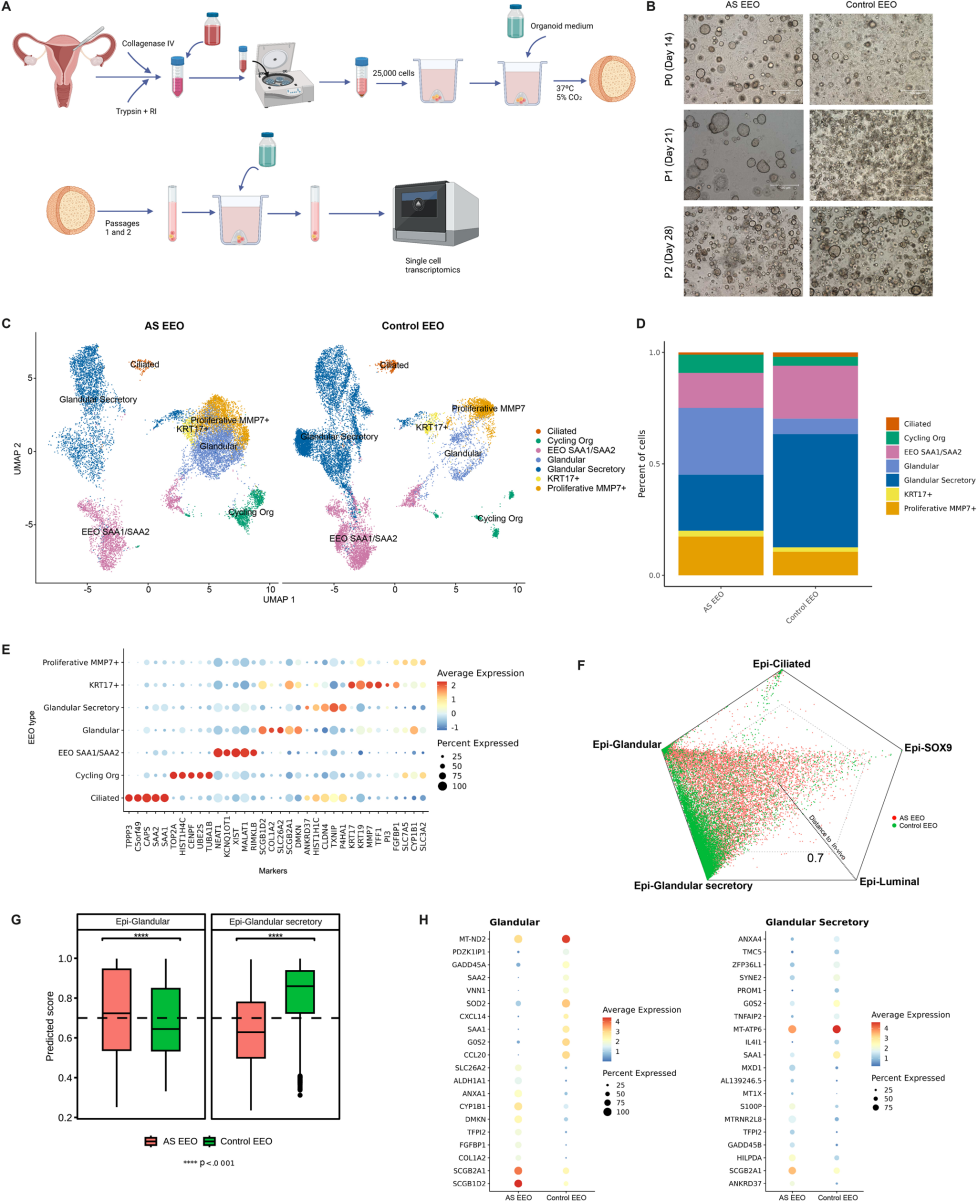
Analysis of endometrial epithelial organoids derived from the window of implantation controls and Asherman´s syndrome patients. **A** Scheme of EEO generation and analysis. **B** Representative images of EEOs generated from AS endometria (AS EEO, *n* = 3) at different passages (P0, 1, 2 = Passage 0, 1, 2) and WOI controls (Control EEO, *n* = 3). **C** UMAP integration of main cell types detected in EEOs derived from AS patient and WOI control endometria (AS EEOs, 14,459 cells; control EEOs, 15,594 cells). **D** Cell type distribution across groups in EEOs generated from AS patient and WOI control endometria. **E** Dot plot displaying the expression of selected genes that distinguish the main EEO cell populations. **F** Polygonal representation of cell-type identity probabilities predicted by a logistic model trained on epithelial cell subtypes from in vivo WOI samples. **G** Box plot prediction scores for each cell type and organoid group. **H** Differential gene expression dot plots for epithelial glandular subtypes in AS EEOs and control EEOs (two-sided Wilcoxon Rank Sum test; FDR < 0.05). Dunn test applied to evaluate differences between groups; two-side Dunn test was applied to evaluate differences between groups; *p*-values were adjusted using the Bonferroni method (*n* = 3 independent samples for each group); **p < 0.01; ****p < 0.0001. AS Asherman’s syndrome, EEO endometrial epithelial organoids, Epi epithelial, P passage.

Finally, we profiled EEOs at the single-cell level to compare this model system with our in vivo data to further evaluate our findings. We collected endometrial samples from three AS patients and three healthy WOI controls. We generated EEOs and passaged them twice to remove stromal cell contamination (Fig. 6A, B).

Single-cell transcriptomic profiling of hormone-treated EEOs acquired data from 30,053 cells (AS 14,459, and WOI controls 15,594). We characterized EEO cell identities by applying a logistic regression predictive model (Methods) and inferring from in vivo data. We projected recovered cells in individual uniform manifold approximation and projections (UMAPs) (Fig. 6C) and identified seven cell populations that matched those from previously characterized epithelial organoids^19^, including glandular, glandular secretory, ciliated, proliferative MMP7+, SAA1/SAA2+, and KRT17+ cells (Fig. 6D, E reports the expression of canonical marker expression).

We calculated a similarity score between our in vivo and in vitro AS model by measuring the distance for each identified cell subtype in the two EEO groups using the in vivo WOI control as a reference. The polygonal plot revealed that glandular epithelial cell subtypes had the highest similarity scores in AS, and WOI control EEOs compared to the in vivo reference (Fig. 6F). When we stratified these scores in boxplots, the transcriptomic profile of AS EEO cells became significantly different from WOI control EEO cells and in vivo counterparts, especially in the glandular secretory epithelium (Fig. 6G, H; Supplementary Data 8).

These results suggest that EEOs represent a useful in vitro model for studying AS.

## Discussion

We report a comprehensive functional single-cell AS endometrial atlas, which describes thirty-two cell identities and a SLPI+ epithelial cell type that characterizes AS during the secretory phase, specifically during the WOI. We revealed significant differences in cell population ratios, gene expression profiles, and CCC networks associated with AS that support a dysfunctional pro-fibrotic, pro-inflammatory, and anti-angiogenic endometrial environment, supporting previous observations^6^. Our single-cell analysis of endometrial samples obtained under hysteroscopic visualization in areas free of IUAs suggests that moderate and severe AS relate to global endometrial dysfunction in which IUAs are the consequence but not the cause.

The AS SLPI+ epithelium represents a disease-associated cell type^33,34^ that overexpresses the *HSPA*1A and *SOCS3* stress-related genes, with the latter controlling *STAT*3-cytokine activation by JAK inhibition^35^. *SOCS3* also became upregulated in severe AS cases alongside the oxidative stress response apoptotic regulator *JUND*^36^. Markers of stress-related signaling in severe disease have been previously connected to epithelial inflammatory diseases such as inflammatory bowel disease^37^.

The increase in myeloid/lymphoid cell types in AS, including macrophages, CD8+ T cells, DN T cells, NK cells, and cytotoxic NK cells, suggests the establishment of a pro-inflammatory state. Macrophages displayed an activated phenotype, dendritic cells possessed an anti-apoptotic and antimicrobial profile, while CD8+ T cells and NKs presented with a cytokine profile composed of *CCL5*, *CCL3*, and *CC3L1*, which targets *CCR1* receptors on macrophages and DCs to mediate their recruitment. Exposure to IL4 (overexpressed by MAIT cells) supports this immune polarization profile^38^. The increased permeability of venous endothelium to leukocyte extravasation through the *ICAM* and *ITGB2* pathways represents another sign of inflammation associated with AS.

We also observed anti-angiogenic signals coexpressed with transendothelial migration signals in the AS endometrium. PV and endothelial AS cell types upregulated the expression of genes encoding anti-angiogenic factors such as *IGFBP3/5/6*; meanwhile, stromal cells overexpressed other anti-angiogenic factors such as *ADAMTS8* and *DCN*^39^.

Fibrosis in the form of IUAs represents a hallmark of AS. Fibrosis is a reactive process that develops in response to epithelial injury and inflammation in which fibroblasts synthesize significant amounts of collagens and proteoglycans, thereby raising tissue stiffness. Our findings associated fibrosis with the massive production of ECM components (collagen, laminin, and *FN1*) by stromal fibroblasts in AS. Analyses of ligand-receptor pair expression to expand our knowledge regarding CCC revealed a shift from epithelial-stromal interactions to ECM-based self-stromal signaling. Furthermore, the AS-associated tissue upregulated the expression of the *ABI3BP* ECM anchor gene^40^ in the stroma and upregulated other pro-fibrotic communication networks such as Periostin signaling between SMC and PV cells^41^, and *FGF2* signaling between EC-vein/glandular epithelium and stroma. Beyond fibrosis, these results may reflect the emergence of tissue adhesion and rigidity that could later alter the invasion phase in abnormal placentation^1^.

We observed a noticeable reduction in the epithelial population in AS patients, especially the ciliated and glandular secretory epithelium essential for WOI function^15^. Disease-associated alterations occurred alongside the downregulation of WOI-associated genes (e.g., *PAEP, GPX3, CXCL14, and MT* family genes), consistent with the dysfunctional receptive epithelium implicated in poor reproductive outcomes in AS patients^42^. In our dataset, we also observed an upregulated *ESR1* and *PGR* expression in the glandular, glandular secretory, luminal epithelial subpopulations, and stromal compartments of the AS endometrium, which suggests the regulation of immune cell inflammatory response by estrogen receptors^43^.

Exploring the WNT pathway revealed the absence of the *WNT7A-FZD6/LRP6* interaction in the SOX9-epithelial cell type in AS. Epithelial cells require the presence of WNT/ncWNT ligands secreted by the stroma to differentiate into luminal cells, while the NOTCH signaling pathway regulates the progression of epithelial cells into the glandular subtype^19^. Our analysis revealed decreased communication between stromal and luminal cells via the ncWNT pathway (W*NT5A-FZD6*), which potentially slows luminal cell differentiation. Furthermore, we observed a decrease in NOTCH pathway (*JAG1-NOTCH2*) communication in glandular, glandular secretory, and luminal epithelium, which corresponds with the dysregulated differentiation of glandular epithelium in AS.

Two major interactors with the WNT pathway – the BMP and Notch pathways – displayed an increase in the AS-stressed epithelium in severe cases through *MSX1* and *MSX2* homeobox genes. These genes represent well-known regulators of the endometrial paracrine signaling pathway^44^ crucial for endometrial organogenesis and receptivity during the WOI.

Besides impaired epithelial differentiation, disconnection of *IL6*, SPP*1, CDH1, DESMOSOME*, and *JAM* pathways prompted detachment across epithelial subtypes, leading to the loss of the epithelial lining in AS. Of note, the stromal decidualization process requires SPP*1*^31^.

We employed endometrial organoids as an in vitro model to recreate the endometrial epithelium of AS patients and then compared organoids created from healthy WOI controls. As self-forming 3D epithelial “mini-organs,” organoids reproduce the functionality and biology of epithelial compartments^45^. Overall, EEOs may represent a useful in vitro model for studying AS and understanding the mechanisms controlling endometrial epithelial regeneration in AS patients.

The limitations of our study include the undetermined impact of AS outside of the secretory phase, which may require the integration of additional data in the proliferative phase. A potential confounding element in this work may arise due to the comparison of AS patients undergoing hormonal replacement therapy (HRT) cycles with control patients undergoing natural cycles; however, HRT cycles and natural cycles are highly reproducible in endometrial maturation and clinical outcomes after embryo transfer^46^. Also, most moderate and severe AS patients do not undergo menstruation, and natural cycles are, therefore, unsuitable for comparison. Additionally, we obtained single-cell EEO results at P2, with a technical enrichment of organoid-forming cells, while the presence of XAV939 (a WNT inhibitor) in the EEO culture media may mask significant differences.

AS profoundly impacts the quality of life and fertility of a non-negligible percentage of women of reproductive age. For the first time, we profiled the cellular and molecular abnormalities present during the secretory phase of AS patients, with particular attention on the WOI.

## Methods

### Subject details

#### AS patients

The study was conducted in accordance with the International Conference on Harmonization Good Clinical Practice guidelines and the Declaration of Helsinki. All procedures involving human endometrium were approved by the Spanish Agency of Medicines and Medical Devices (Agencia Española de Medicamentos y Productos Sanitarios [AEMPS]) (April 20, 2020) and the Clinical Research Ethics Committee at the Hospital Universitari Vall D’Hebron Barcelona, Spain (April 17, 2020). All participants provided written informed consent for sample collection as well as to use their information for publication purposes.

Nine patients of 34–42 years of age diagnosed with moderate and severe AS were enrolled according to the American Fertility Society (AFS) classification of IUAs^47^. The diagnosis of AS was confirmed by the same surgeon (X.S.) that performed all hysteroscopies in the secretory phase. All patients were treated with HRT to synchronize cycles and treatments, and hysteroscopies were always performed during the WOI period, as determined in each AS patient by the transcriptomic signature of endometrial receptivity using a commercially available endometrial receptivity analysis (ERA) test^48^. Patients received 6 mg estradiol valerate daily (starting on the second day of menstruation) for 10–12 days. After this period, 400 mg of natural micronized progesterone was administered vaginally every 12 h for 4–6 days, according to the ERA results before the endometrial biopsy.

Endometrial biopsies were obtained via hysteroscopic vision (Betocchi 5 mm, Karl-Storz, Germany) using a semi-rigid double-action biopsy and grasping forceps (Karl-Storz, Germany). Samples were obtained from the posterior wall of the uterus at least 1 cm away from any visually identified IUA under direct vision.

The endometrial microbiome was also assessed in AS patients, which ruled out the existence of active endometritis that may affect typical endometrial tissue composition.

Requirements for participation in the study included a patient age of 18–44 years old, a BMI of 18–30, normal liver, heart, and kidney function, the absence of pregnancy, HIV, Hepatitis B or C, syphilis, or any psychiatric pathology, and a willingness to complete the study.

All nine patients (# 5, 7, 8, 9, 10, 12, 13, 14, and 15) underwent endometrial biopsy during the WOI for single-cell transcriptomics. For organoid studies (see below), samples from the last three patients (#13, 14, and 15) were used.

#### Secretory phase controls

Single-cell transcriptomes from ten fertile women as controls of normal secretory endometrium were analyzed (available at GSE111976 dataset^15^) (Supplementary Fig. 1).

#### WOI controls

The WOI control group comprised single-cell data from six single-cell transcriptomes obtained during the WOI from the GSE111976 dataset and six endometrial samples collected during the WOI from healthy donors.

Endometrial biopsies of control individuals at the WOI were collected from fertile and asymptomatic women during the WOI (See GA#874867 in Supplementary Fig. 1 and Supplementary Data 1) following the same clinical protocol and sample collection described in Wang et al.^15^. Ethical approval was given by the Research Ethics Committee of the University of Tartu (approval reference 302/T-4). All participants provided written informed consent.

Endometrial samples were fixed with paraformaldehyde and embedded in paraffin for hematoxylin and eosin (H&E) staining or used for single-cell analysis. Representative images of endometrial biopsies from AS patients (*n* = 9), and control biopsies from healthy patients are shown in Supplementary Fig. 2A, B.

### Processing and dissociation of endometrial biopsies

A two-stage dissociation protocol separated endometrial biopsies into stromal fibroblasts and epithelial-enriched single-cell suspensions^15^. Before dissociation, tissue was rinsed with phosphate-buffered saline (PBS) in a petri dish to remove blood and mucus, and excess PBS was removed after rinsing. The tissue was minced into small pieces and dissociated with 3 ml collagenase mix (Collagenase V, Sigma), RPMI + 10% fetal bovine serum, and DNaseI (Sigma) at 37 °C under continuous shaking at 175 rpm for 25 min in a 15 mL Falcon tube in a horizontal position. This primary enzymatic step mostly dissociates stromal fibroblasts into single cells, leaving epithelial glands and lumen undigested. The contents were transferred to a 50 mL tube in RPMI media and filtered with a 100 μm cell strainer. The tissue remaining on the filter was used for the epithelial enrichment cell isolation by resuspending and incubating in 10 ml Trypsin mix (Trypsin -EDTA (0.25%) phenol and DNaseI) for 10 min.

The resulting two contents were transferred to 50 mL tubes with 20 mL RPMI, filtered with a 100 μm cell strainer, centrifuged, and resuspended in 1 mL RPMI. Dead cells were removed using the MACS dead cell removal kit (Miltenyi Biotec). Live-cell suspensions were loaded into a Chromium Next GEM Chip G (10X Genomics) to obtain cDNA from individual cells.

Mean cells obtained in stromal and epithelial-enriched suspensions were 2.38 × 10^6^ (5 × 10^5^−6.4 × 10^6^) and 1.25 × 10^6^ (2 × 10^5^−5.3 × 10^6^), respectively. The viability of stromal cells was 67.44% (31–87%) and 39.5% (20–80%) for epithelial cells.

### 10X Genomics Chromium library preparation and sequencing

Total RNA was extracted using the RNeasy Micro Kit with on-column DNase treatment (Qiagen) following the manufacturer’s instructions. RNA was resuspended in 14 μl of RNase-free water, and purity and concentration determined using a NanoDrop 1000 spectrophotometer (Thermo Fisher). For cDNA synthesis, 500–1000 ng of total RNA was reverse transcribed following the manufacturer’s instructions (Thermo Fisher).

10X-Genomics v3 libraries were prepared as per the manufacturer’s instructions. Libraries were sequenced, aiming at a minimum coverage of 50,000 raw reads per cell, on an Illumina NovaSeq 6000 S2v1.5 300 cycles at a mean 40x coverage (paired-end; read 1: 26 cycles; i7 index: 8 cycles, i5 index: 0 cycles; read 2: 98 cycles).

### Alignment and quantification of gene-to-cell count matrices from 10X scRNA-seq

scRNA-seq data was processed with the CellRanger software suite (version 3.1.0). Raw reads were demultiplexed using the *mkfastq* wrapper command of *bcl2fastq* (Illumina). The libraries were mapped using GRCh38-3.0.0 as a reference provided by 10X Genomics. The count gene expression matrices per cell were computed per sample using the counts pipeline (with *--expect-cells* parameter set to default), which performs the steps of read alignment (with STAR mapping tool), UMI counting, calling of cell barcodes, and filtering of empty droplets based on a simple Good-Turing smoothing model of background gene expression profiles allows the discrimination of low RNA content cells from ambient RNA.

### Quality control cell filtering and doublet detection

Low-quality cells in samples were filtered out using the distributions of the detected number of genes, detected the number of counts, and mitochondrial percentage of read counts. Cells with 1 median absolute deviation (MAD) in at least two conditions were filtered out from the sample. These quality control and downstream analyses were performed in R version 4.1.

Doublet detection was performed with two methods: *DoubletFinder* (2.0.3R package) and *scds* (1.6.0R package). The expected doublet formation rate was fixed for each sample according to the cell count recovery given per sample by CellRanger. The hybrid approach from the *scds* package was used to avoid removing false-positive doublets. Cells marked as doublets by both algorithms were removed from the samples.

To evaluate how much expression of WOI genes was coming from ambient RNA, and to be able to highlight them in cell populations outside of the epithelium compartment, we utilized the CellBender (0.2.2) tool on the raw count matrices, as generated by the CellRanger pipeline. An examination of Cell Ranger’s Barcode Rank Plot for each sample allowed the configuration of CellBender^49^ to account for barcodes associated with empty droplets (containing ambient RNA). The ambient plateau was selected, which consists of barcodes below the plot knee - with relatively low counts - for each sample. The false discovery rate (FDR) value was also set to 0.05. The contaminating counts (the remainder between corrected vs. uncorrected) were then utilized to compute the comparison between the AS and WOI control samples, leading to the identification of a specific gene set signature associated with ambient RNA. These results are represented by the percentage of reads of ambient RNA contamination present in each gene that are filtered out by CellBender (Supplementary Data 6 and  9).

### Integration of scRNA-seq data across conditions and cell clustering

Individual samples were aggregated, processed, and integrated with functions from the Seurat package (4.0.1). First, sample-to-sample aggregation for each condition dataset was performed with the *merge* function. Three aggregated datasets were constructed: (i) AS samples, (ii) control samples collected in our previous single-cell study of the natural menstrual cycle (GSE111976)^15^, and (iii) control samples at WOI (GA#874867). The resultant count matrices were further processed to exclude cells with fewer than 750 detected genes and under a maximum mitochondrial content of 25%.

Each aggregated dataset was processed with the *SCTransform* function to integrate with the dataset of contrast to approach two integration analyses: (i) AS samples collapsed with secretory control samples, and (ii) AS samples collapsed with WOI control samples. The *SCTransform* pipeline integrates different study conditions by applying a regularized negative binomial regression to allow the detection of condition-to-condition shared cell identities and the performance of differential expression analysis.

Mitochondrial ratios and cell cycle phase were regressed out from integrated datasets. *SelectIntegrationFeatures* was used to select the variable genes to integrate. *PrepSCTIntegration* and *FindIntegrationAnchors* were applied to identify the anchoring vectors across datasets and integration steps performed with *IntegrateData* (normalization method set to SCT). In the dimensional reduction step, the first thirty dimensions of principal component analysis (PCA) and UMAP were used to visualize cell clusters on the nearest-neighbor graphs.

Recoverable cells were manually labeled with the cell populations detected in integrated AS/control datasets to detect cell types unique to the disease condition. These labels were downstream transferred from one dataset to the other by the anchor integration protocol in the following analysis steps.

### AS cartography: integration by the dataset of origin

Control samples were retrieved from the 10X Single-cell 3’v3.1 dataset (available at GSE111976). Cell populations detected in AS were aggregated with retrieved control cells. The Seurat Integration protocol was applied to integrate the two datasets. Raw Seurat objects of control and AS samples were split into two objects. The *SCTransform* function processed each object to processing counts. The dataset was further integrated using the 3000 most variable genes. *IntegrateData* was applied using the dataset of origin as an integration factor. The integration step was validated by evaluating the conservation of canonical markers across AS and control conditions (Supplementary Figs. 3A, B and 4A, B; Supplementary Data 2, 3, and 4).

### Annotation of cell types in scRNA-seq datasets

The main cell types were identified by examining cluster-specific differentially expressed gene(s) compared to other clusters. The Wilcoxon Rank Sum test was used for each cluster differential expression, and the p-values were adjusted using the FDR method. The first approach determined cluster annotations by contrasting cluster-specific differentially expressed genes against accessible biological information. The main annotated cell types and their descriptive gene markers are available in Supplementary Data 3. In a second approach, aiming to annotate the cellular subtypes of the WOI at finer resolution, the anchor transfer labels protocol from Seurat was applied to propagate more granular identities from two recently published endometrial atlases^17,19^. Only identities scoring over 0.7 were considered for later annotation steps. Then cell types were re-evaluated, and clusters with new consistent transferred labels and marker profile expression were manually reannotated.

### Analysis of differential cell abundance

Cell ratio analysis across different experimental groups was performed using the NB-GLM function from the edgeR (3.34.0) package. A count matrix of the different cell types detected per sample was created and used as input to compute the differential abundance (DA) tests across cell types. The FDR *p*-value adjusting method was applied. Cells detected as cycling cells were removed prior to differential abundance analysis. All cell type ratios of AS vs. control are available in Supplementary Data 10.

### Differential expression

The *FindMarkers* function from the *Seurat* package was used to perform differential expression analysis between AS and control samples (Supplementary Data 5 and 6) and between cell types reported in the dataset (Supplementary Data 2, 3, 4, and 6). The Wilcoxon Rank Sum test was used, and the *p*-values were corrected using the FDR method. Genes with FDR values under 0.05 were considered significant.

In EEOs, differentially-expressed genes in AS vs. controls shared with the same comparison in vivo for glandular and glandular secretory populations were shortlisted to identify genes consistently regulated across conditions.

### CCC network analysis

Analyzing the potential signaling interactions between cells, we used two metrics provided by the *CellChat* (1.1.3) package: the total interaction probability and the communication information flow. The total interaction probability measurement indicates the likelihood of communication between two cell types. This measure decomposes the interaction probability between a sender and a receiver cell type based on the number of interactions (L-R pairs expressed) and the interaction’s strength (level of expression). The database used to mine interaction information comprises a curated list of molecules grouped into 229 signaling pathway families, which considers the known structures of ligand-receptors, including multimeric complexes, soluble agonists and antagonists, and both stimulatory and inhibitory co-receptors. The measure of communication information flow assesses the amount of information exchanged between multiple cells through multiple interactions. A higher value of communication information flow among cell populations indicates a more intricate communication network. To correct the influence of each cell population, and remove *cycling* cells from downstream analysis, the population.size argument in the *computeCommunProb* function was used. CCCs were filtered to exclude those with less than ten cells. For network differential testing between AS and control groups, the *ranknet* function was applied with a significance threshold of 0.05. Only common cell types could be included in group comparisons. The information flow bar charts highlight significant signaling pathways between groups, which can be further examined through chord plots of interaction probability to identify the receiver and sender cells. The contribution of each L-R pair to a pathway is also displayed in bar plots of interaction probability. Supplementary Data 7 comprehensively reports all significant pathways. The source data to reproduce all CCC networks are included as Supplementary Data 11 and 12.

### In situ RNA hybridization validation

In situ RNA hybridization used the Advanced Cell Diagnostics RNAscope® Multiplex Fluorescent Detection kit v2 (Bio-techne) according to the manufacturer’s instructions. First, 5 μm Formalin-Fixed Paraffin-Embedded (FFPE) human endometrial specimens were placed into SuperFrost Plus slides (VWR) and baked in the HybEZ hybridization oven (Advanced Cell Diagnostics) at 60 °C for 1 h to dry and ensure adhesion. To deparaffinize FFPE sections, samples were rinsed in Histo-Clear I for 5 min at room temperature (RT), followed by Histo-Clear II for 5 min, washed with fresh 100% ethanol at RT, and dried at 60 °C for 5 min. Samples were treated with RNAscope® hydrogen peroxide for 10 min at RT and immediately washed with distilled water. Slides were incubated for 15 min in 1X RNAscope Target Retrieval Reagent preheated at 95 °C in a steamer, washed in distilled water, incubated in 100% ethanol for 3 min, and dried at RT. Sections were subsequently incubated with a protease solution (RNAscope® protease plus) for 30 min at 40 °C in the HybEZ hybridization oven. Target probes for HS-SLPI-C1 *Homo sapiens* SLPI were applied to the sections, incubated at 40 °C for 2 h, and washed twice for 2 min in washing buffer solution before amplification. Sections were then incubated with amplifier probes by applying Multiplexv2 AMP1 (40 °C for 30 min), Multiplexv2 AMP2 (40 °C for 30 min), and Multiplexv2 AMP3 (40 °C for 15 min). Fluorescent signal was obtained by incubating the samples with RNAscope Multiplex FLv2 HRP-C1 for 15 min, the respective fluorophore for 30 min (SLPI associated to TSA Vivid™ Fluorophore kit 520 nm (Cat. #7523) at a 1:750 dilution, and then RNAScope Multiplex FL v2 HRP Blocker for 15 min. Slides were then incubated in DAPI for 30 s to stain nuclei and then mounted using Prolong® Gold Antifade Reagent (Life Technologies, Cat. #P36934). RNAscope 3-Plex Negative Control Probe (Cat. #PN 320871) and RNAscope 3-Plex Positive Control Probe (Cat. #PN320861) were used as controls for target probe imaging and to assess background signals. Fluorescent images were captured using a 63x oil objective using a Leica TCS-SP2-AOBS Confocal microscope. Images containing single or clustered dots were interpreted as positive and those without as negative for the target RNA.

### Organoid culture from endometrial biopsies

Organoids were generated from AS patients’ endometrium (*n* = 3) and from healthy control (*n* = 3) endometrium following the protocol described by Boretto and collaborators^16^ with minor modifications. After enzymatic dissociation of the endometrial biopsies (described above), epithelial and stromal cells were obtained (in the same tube), and the total cell numbers were counted. After centrifugation at 300 g, the pellet was resuspended in 70% Matrigel (Corning, Cat. #356231) and 30% DMEM/F12 (Thermo Fisher Scientific, Cat. #11330032) supplemented with 2 µM rho-associated protein kinase inhibitor Y-27632 (Merck, Cat. #SCM075). The suspension was cultured in 20-µl droplets containing 25,000 cells, deposited in prewarmed 48-well plates, and allowed to form a gel at 37 °C and 5% CO_2_ before adding culture medium. Organoid culture medium was prepared as previously described (Supplementary Table 1)^16,50^. Matrigel droplets were cultured for 14–16 days in the first passage (P0) with medium changes every two days. Organoids were then grown for seven days before the following passage, with media changes every 2 days.

Organoids were recovered for passaging by liquifying Matrigel droplets with ice-cold DMEM/F12 (without any growth factors, serum, or enzymes), followed by repeated pipetting to ensure maximum recovery. Organoids were then dissociated by incubation with TrypLE for 5 min at 37 °C supplemented with 1 µM Y-27632 and mechanically triturated. The resulting cell suspension was centrifuged at 300 × *g*, and the pellets were resuspended in 70% Matrigel and 30% DMEM/F12 supplemented with Y-27632 and placed as droplets in 48-well plates. Two passages (till P2) were performed to remove the contaminating stromal cells from the epithelial organoids. For hormonal treatment, P2 organoids were plated at 15,000 cells per well in a 48-well plate and treated with steroid hormones after two days of culture with organoid medium (Supplementary Table 1), as previously described^51^. Briefly, organoids were first cultured with 10 nM 17β-estradiol (E2) (Sigma Aldrich, Cat. #E2758) for 2 days. Then, the media was supplemented with 10 nM E2, 1 µM medroxyprogesterone 17-acetate (MPA) (Sigma Aldrich, Cat. #M1629), 0.25 mM 8-bromoadenosine 3′, 5′-cyclic monophosphate sodium (cAMP) (Merck, Cat. #B7880), and 10 µM XAV939 (Selleckhem, Cat. #S1180) for four additional days. After hormonal treatment, organoids were dissociated for single-cell transcriptomic analysis following the same protocol described for passaging but with an additional cell counting step for the single-cell transcriptomics protocol (10X Genomics, described above).

### Identifying organoid cells using machine learning

The transcriptomic identity of organoid-derived cells was inferred using their relative in vivo cell subtypes following a similar approach as that described by La Manno et al.^52^. Detected epithelial cells from the GSE111976 dataset were used, and the different subpopulations were studied. Previously described gene cell markers were used to label cells^15,19^.

Before training the model, a feature selection of genes was performed. First, the top 4000 most variable genes were detected using the *FindMarker* function from Seurat. Second, the specific gene markers of each cell type were computed using only the previously selected gene set. Finally, the top 1000 genes ranked by three metrics of cell type specificity were selected (fold-increase, fold-increase*fraction-positive, and fold-increase*fraction-positive^0.5). A machine learning logistic regression model (from *caret* (6.0-92) and *glmnet* (4.1-4) R packages)^53^ was then used to predict the identity of organoid cells. The training dataset was split randomly, with 70% of cells used for training and 30% for evaluation in the testing phase. Fine-tuning of the logistic regression hyperparameters was conducted as follows: cross-validation of ten iterations with the lambda value changed from 0.001 to 0.01 in steps of 0.01. Alpha was held constant at a value of 1. The optimal lambda value was 0.001, and a 0.9446 accuracy (0.9402, 0.9488 95% CI) in the test phase was achieved. Finally, the probabilities of the inferred cell types of organoids were plotted in a polygonal projection.

Finally, the differences between cell type probabilities across experimental groups were evaluated by applying the Kruskal–Wallis test following Dunn’s multiple comparison test. *P*-values were corrected using the Bonferroni method. Differential expression in organoid cells was assessed by applying the Wilcoxon Rank Sum test (FDR < 0.05) as previously described. Results are supplied in Supplementary Data 8. Investigating the transcriptomic similarities between EEO cells and in vivo cells utilized the Harmony (version 0.1.1) and scPred (version 1.9.2) R packages.

### Statistics and reproducibility

Statistical analyses were performed in software R (4.1.2) and no randomization was required since the clinical trial was one-arm study. Moreover, the investigators were not blinded during experiment and outcome assessment. Reproducibility was confirmed by independent experiments, and each specific statistical test and its potency has been described in the associated Figure legends.

### Reporting summary

Further information on research design is available in the Nature Portfolio Reporting Summary linked to this article.

### Supplementary information

Supplementary Information

Description of Additional Supplementary Files

Supplementary Data 1

Supplementary Data 2

Supplementary Data 3

Supplementary Data 4

Supplementary Data 5

Supplementary Data 6

Supplementary Data 7

Supplementary Data 8

Supplementary Data 9

Supplementary Data 10

Supplementary Data 11

Supplementary Data 12

Reporting Summary

### Source data

Source Data

## Data Availability

The single-cell RNA-sequencing data generated for this manuscript has been uploaded to GEO under accession numbers GSE215968 and GSE216748. The GSE215968 dataset contains the in vivo endometrial samples, including AS patients, and control endometrial samples collected under the HUTER project (GA#874867) during the WOI. The GSE216748 dataset includes all EEO samples. Control samples of ten healthy endometrial biopsies during the secretory phase were downloaded from GEO: GSE111976^15^. Source data are provided with this paper Other data that support the findings of this study are available from Asherman Therapy SL. Restrictions apply to data access with data used under license for the current clinical study and are not publicly available. Data are, however, available from the authors upon reasonable request and with permission of the Vall Hebron Ethical Committee.
