# Supplementary material for: Decoding the endometrial niche of Asherman’s Syndrome at single-cell resolution
Source: Nat Commun. 2023 Sep 21;14:5890. doi: 10.1038/s41467-023-41656-1 (PMC10514053; doi:10.1038/s41467-023-41656-1)
Supplement: Supplementary file 1 — Supplementary Information [file 41467_2023_41656_MOESM1_ESM.pdf]

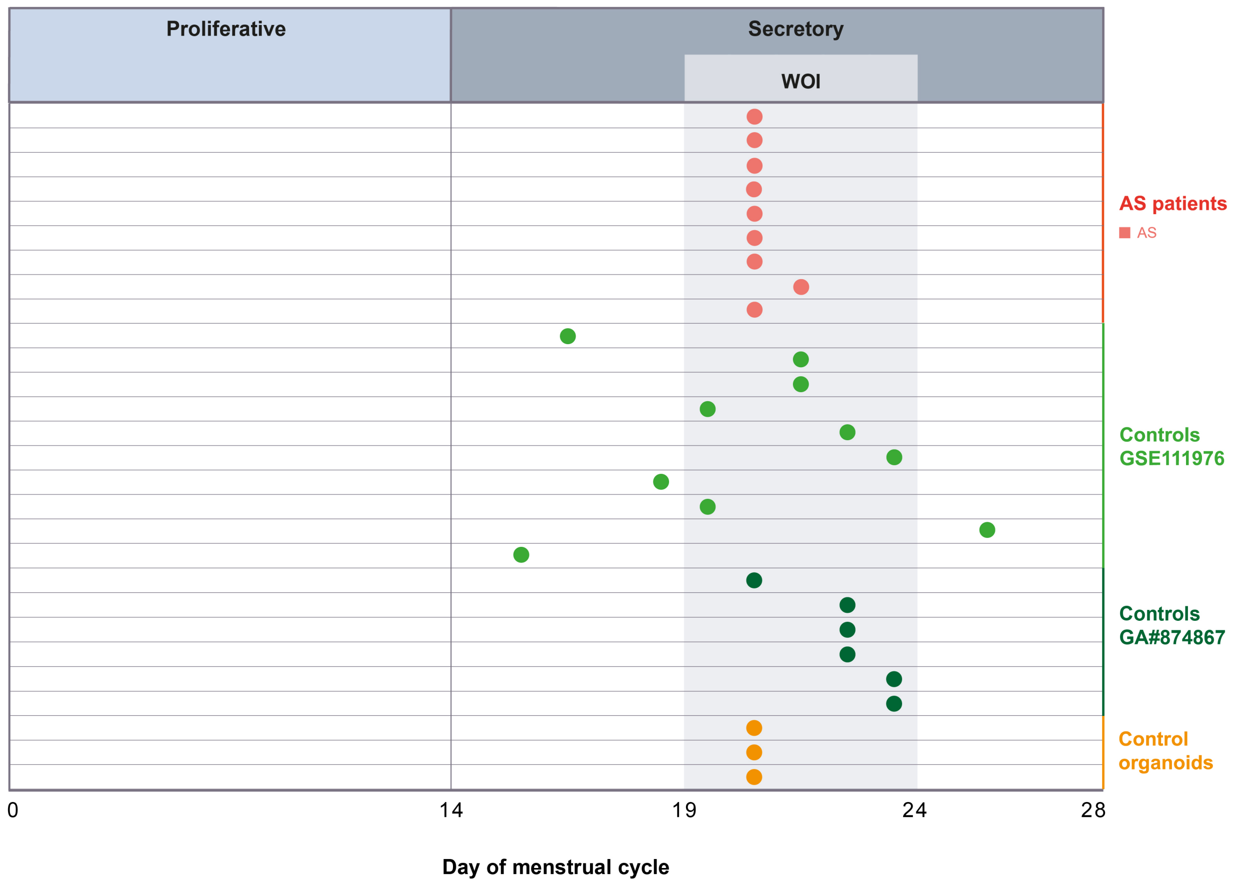

Supplementary Figure 1. Diagram representing the days of the menstrual cycle and endometrial phase of all individuals (patients and controls) analyzed using single-cell transcriptomics. Colored dots represent each patient included in the different datasets in the study. Abbreviation: AS: Asherman's syndrome; WOI: window of implantation.

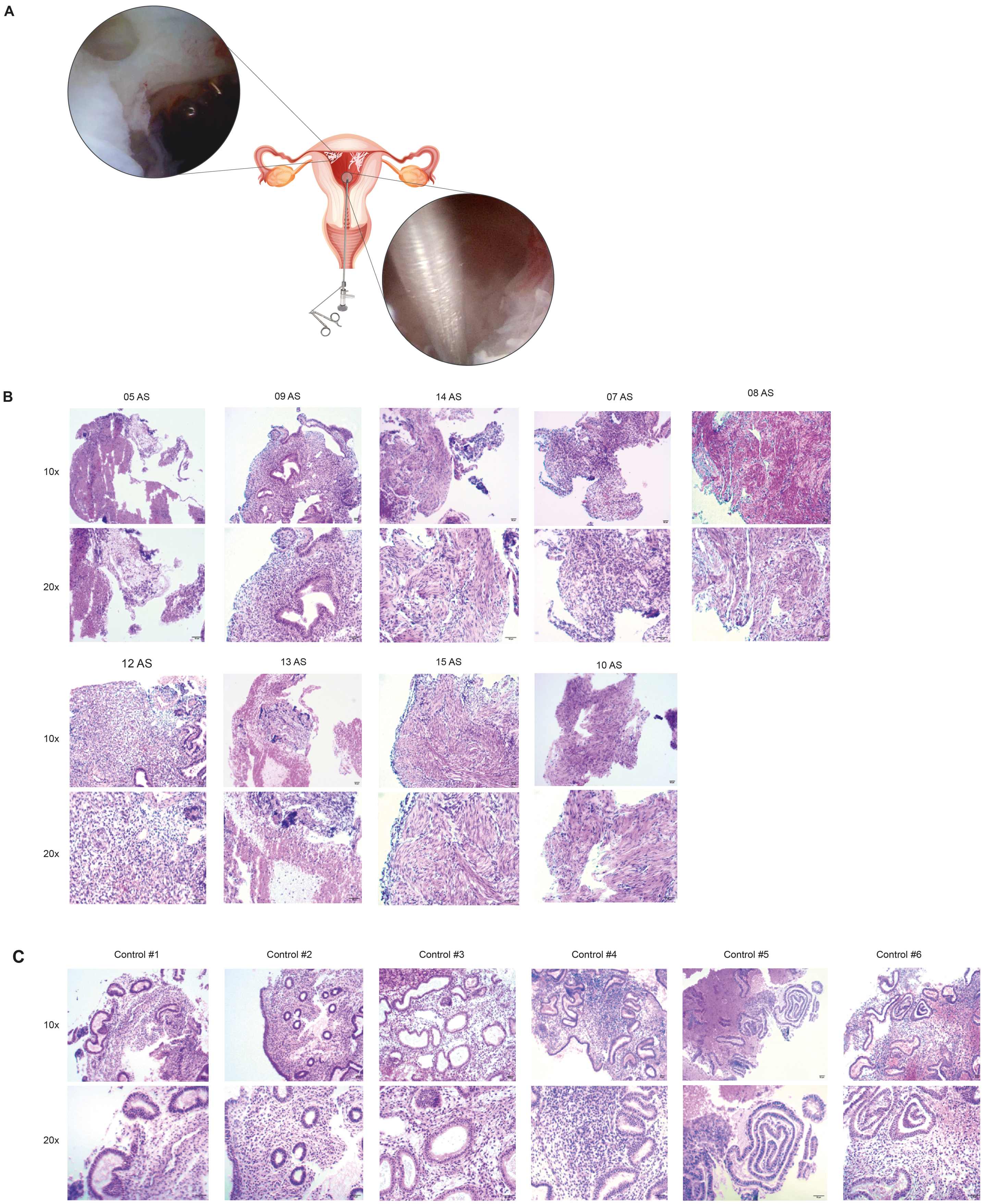

Supplementary Figure 2. A) Schematic diagram of the sample collection procedure. Once the endometrial cavity was assessed by hysteroscopy, endometrial biopsy was obtained with hysteroscopic graspers from the posterior wall of the uterine cavity ≥ 1 cm apart from any intrauterine adhesion. **B)** H&E staining of AS patients (AS) at 10X and 20X magnification (n=9 for independent AS samples before (AS) and after (AS-Post)). **C)** H&E staining of control samples (GA#874867 set) at 10X and 20X magnification. Scale bar = 50 µm for both magnifications. Abbreviation: AS: Asherman's syndrome (n=6 for independent control samples).

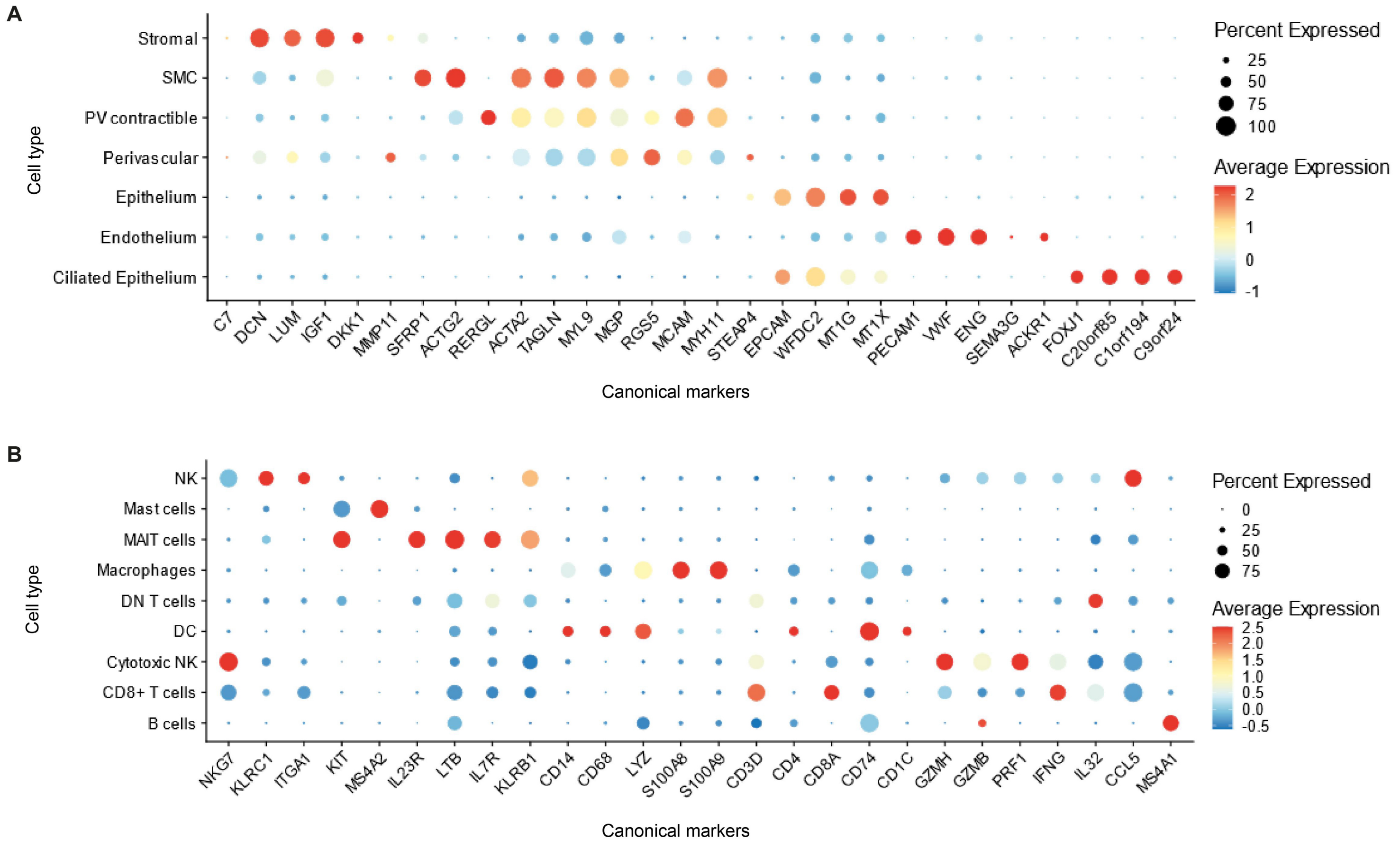

Supplementary Figure 3. Dot plot of canonical marker gene expression in defined A) main populations and **B)** immune populations of the AS cartography (**Supplementary Table 2** contains a complete list of reference canonical markers used to evaluate cell identities). Abbreviations: DC: dendritic cells; DN: double negative; MAIT: mucosal-associated invariant T; NK: natural killer; PV: perivascular; SMC: smooth muscle cells.

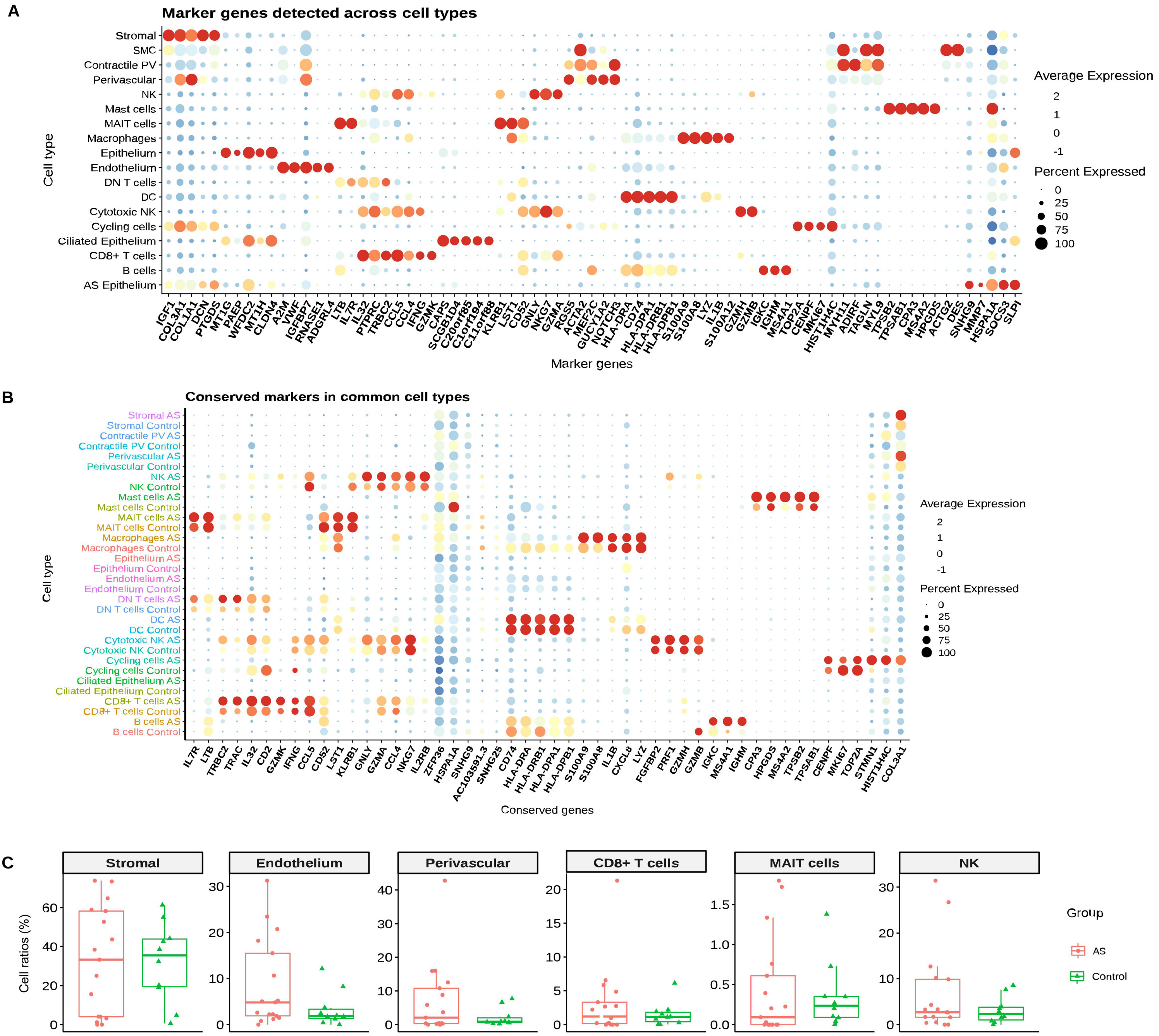

Supplementary Figure 4. A) Dot plot showing the top five differentially-expressed genes in annotated main cell types in the AS cartography. Supplementary Table 3 contains a complete description of differentially-expressed genes. **B)** Dot plot showing the expression of the top five conserved genes between cell types from AS and control endometria (**Supplementary Table 4** contains a complete description of conserved genes). **C)** Differences in cell ratios comparing AS and control endometria (n=9 for independent AS patients, n=10 for independent healthy endometrium patients). Abbreviations: AS: Asherman; DC: dendritic cells; DN: double negative; MAIT: mucosal-associated invariant T; NK: natural killer; PV: perivascular; SMC: smooth muscle cells; UMAP: Uniform manifold approximation and projetion.

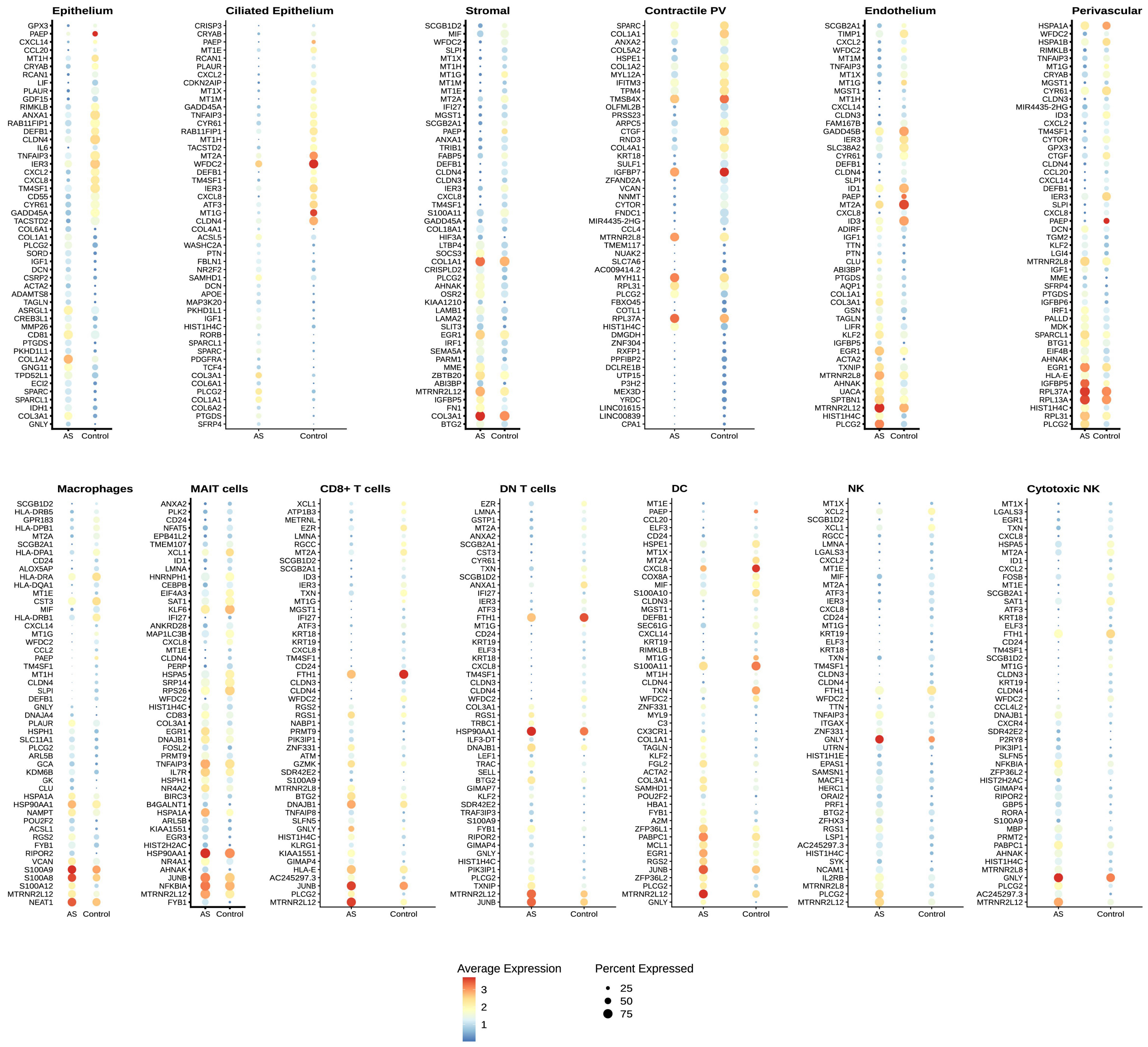

Supplementary Figure 5. Differential gene expression between AS and control endometria separated by cell type (two-sided Wilcoxon Rank Sum test, FDR < 0.05). Abbreviations: AS: Asherman's syndrome; DC: dendritic cells; DN: double negative; NK: natural killer; PV: perivascular.

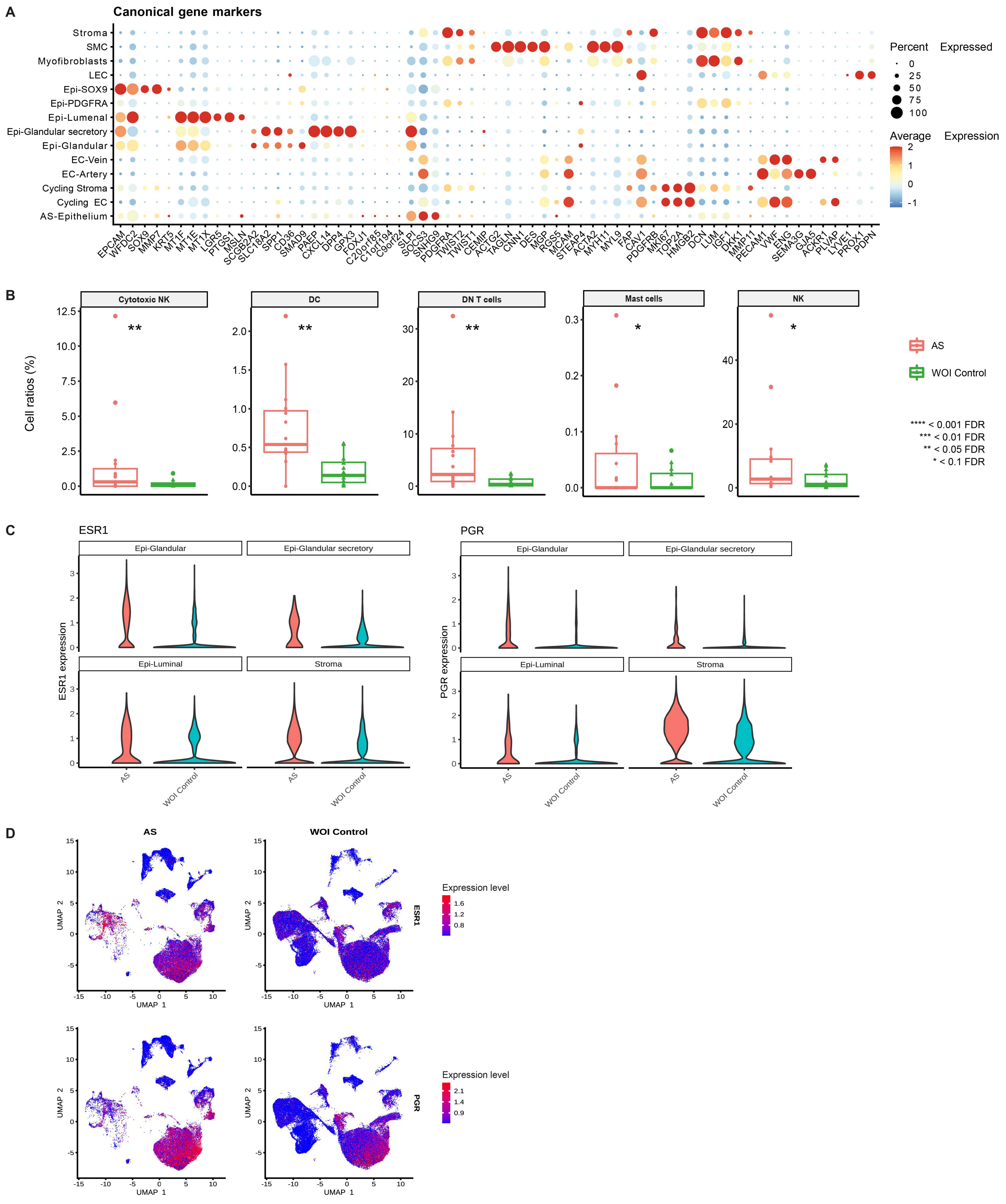

Supplementary Figure 6. A) Dot plot of canonical marker gene expression in AS patients integrated with WOI control endometria. **B)** Cell ratios comparing the endometrium of AS patients to WOI controls (two-sided NB-GLM statistical test; n=9 for independent AS patients, n=12 for independent WOI control samples). **C)** Expression level of ESR1 and PGR in the epithelial and stromal compartments during the WOI in AS vs. control (two-sided Wilcoxon test; FDR < 0.05). **D)** Distribution of ESR1- and PGR-expressing cells during the WOI in AS vs. control. Abbreviations: AS: Asherman; DC: dendritic cells; DN: double negative; EC: Endothelial cell; Epi: epithelial; FDR: false discovery rate; LEC: lymphatic endothelial cell; NK: natural killer; SMC: smooth muscle cells; WOI: window of implantation.

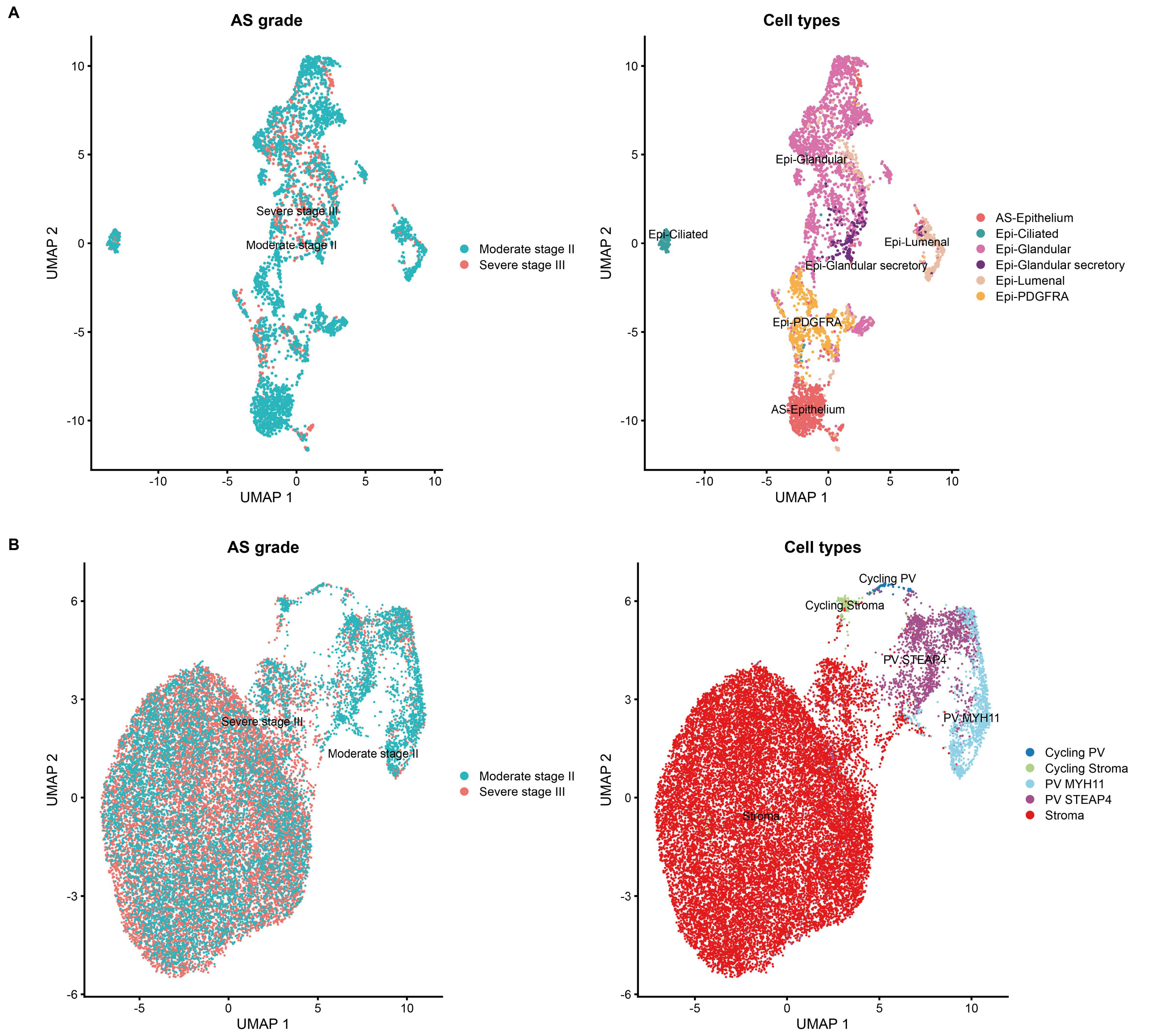

Supplementary Figure 7. scRNA-seq analysis of Asherman´s syndrome endometria classified by severity. A) UMAP of detected epithelial subpopulations. **B)** UMAP of detected stromal subpopulations. Abbreviations: AS: Asherman; Epi: epithelium; PV: perivascular; UMAP: uniform manifold approximation and projetion.

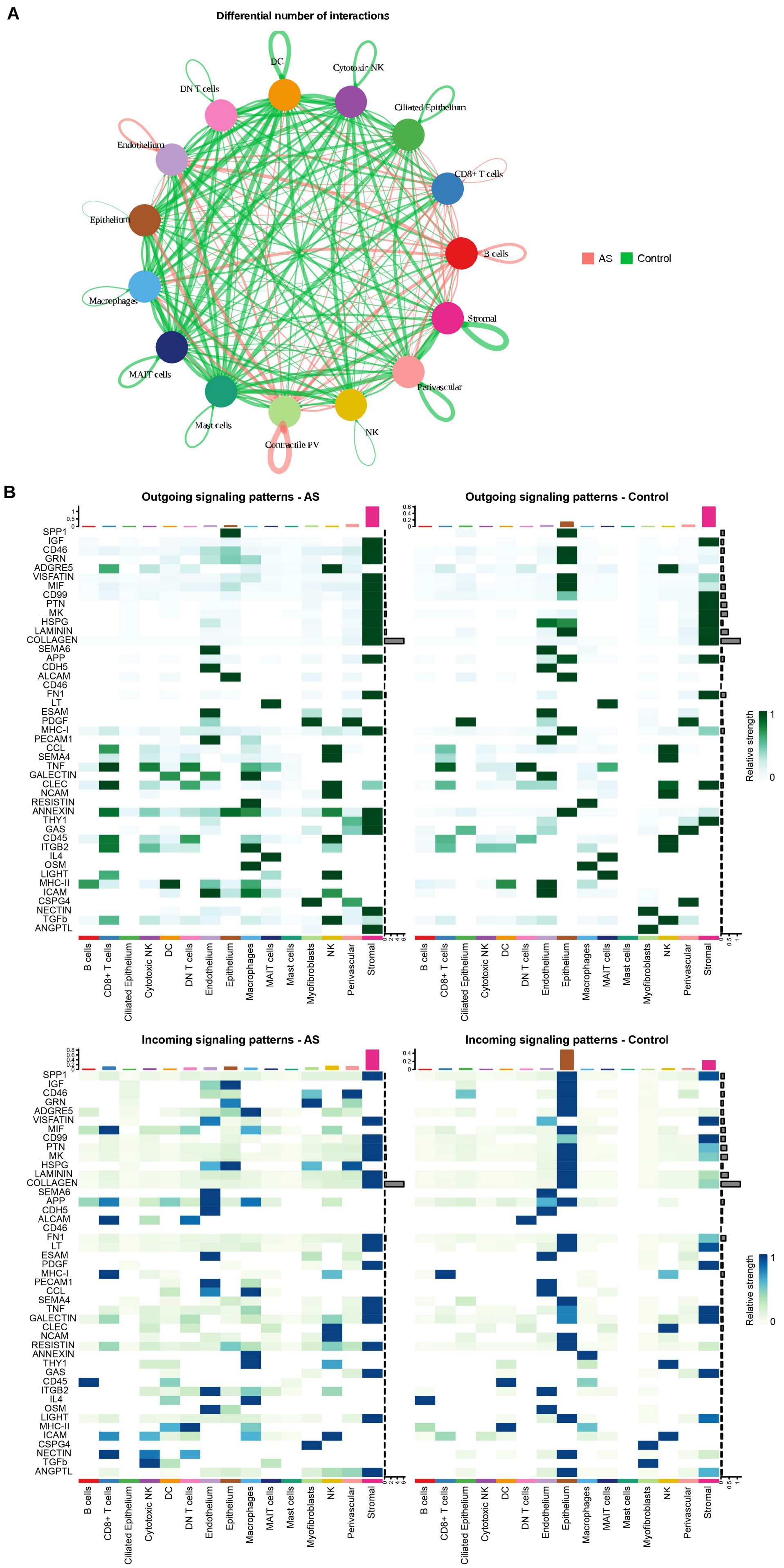

Supplementary Figure 8. A) CCC communication network showing significant ligand-receptor interactions in AS and Control endometria samples. **B)** Heatmap of signaling patterns (outgoing: upper panel, incoming: lower panel) between cell types in AS and Control endometria. Abbreviations: AS: Asherman; DC: dendritic cells; DN: double negative; MAIT: mucosal-associated invariant T; NK: natural killer; PV: perivascular; SMC: smooth muscle cells.

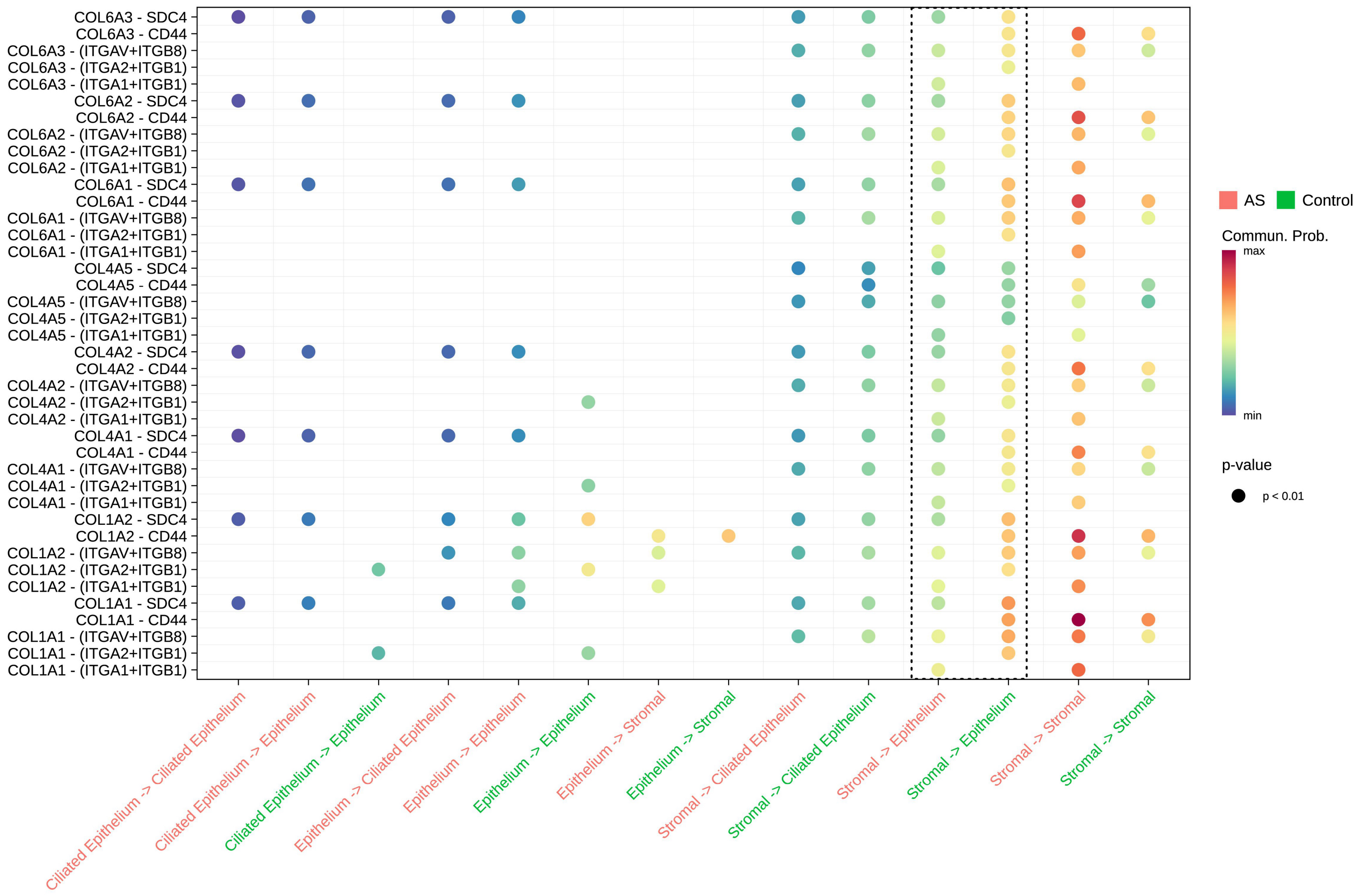

Supplementary Figure 9. Dot plot of CCC communication results for the COLLAGEN signaling pathway (two-sided Wilcoxon test). (AS in red labels, Control in green labels). Abbreviations: AS: Asherman.

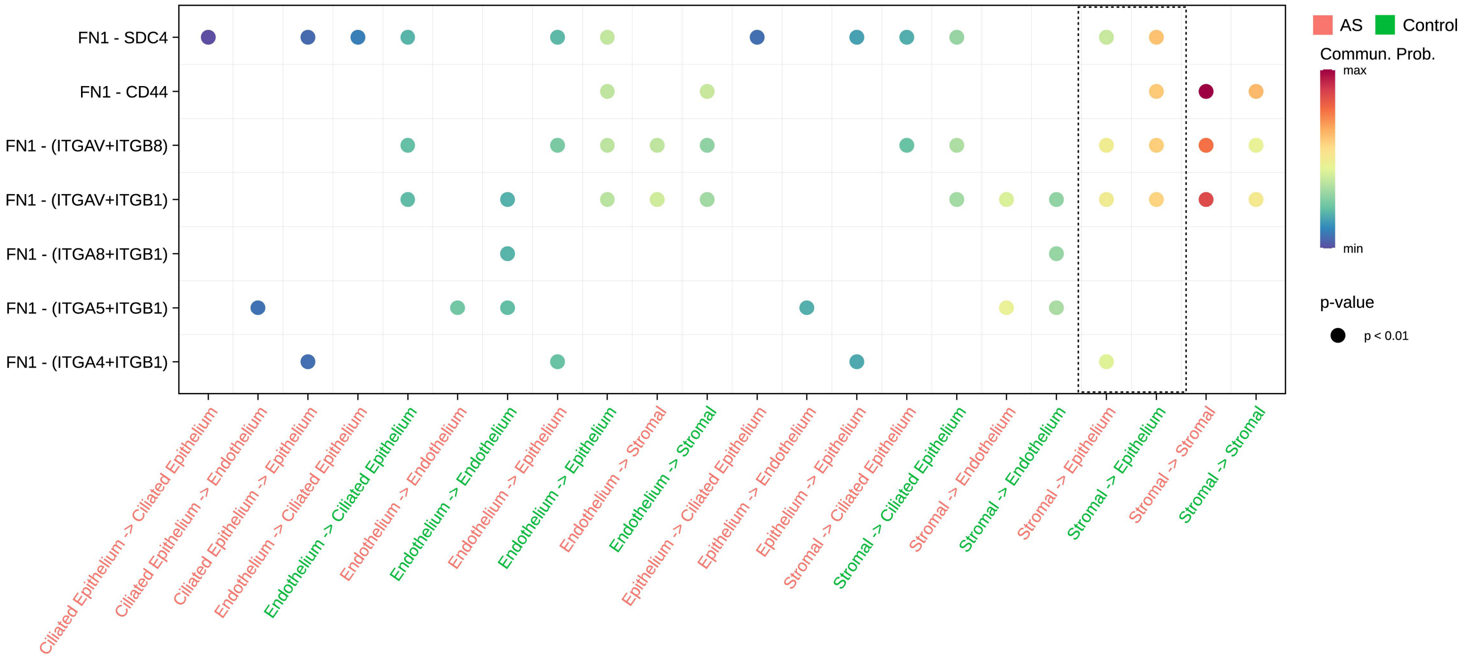

Supplementary Figure 10. Dot plot of CCC results for the fibronectin (FN1) signaling pathway (two-sided Wilcoxon test). (AS in red labels, Control in green labels). Abbreviations: AS: Asherman.

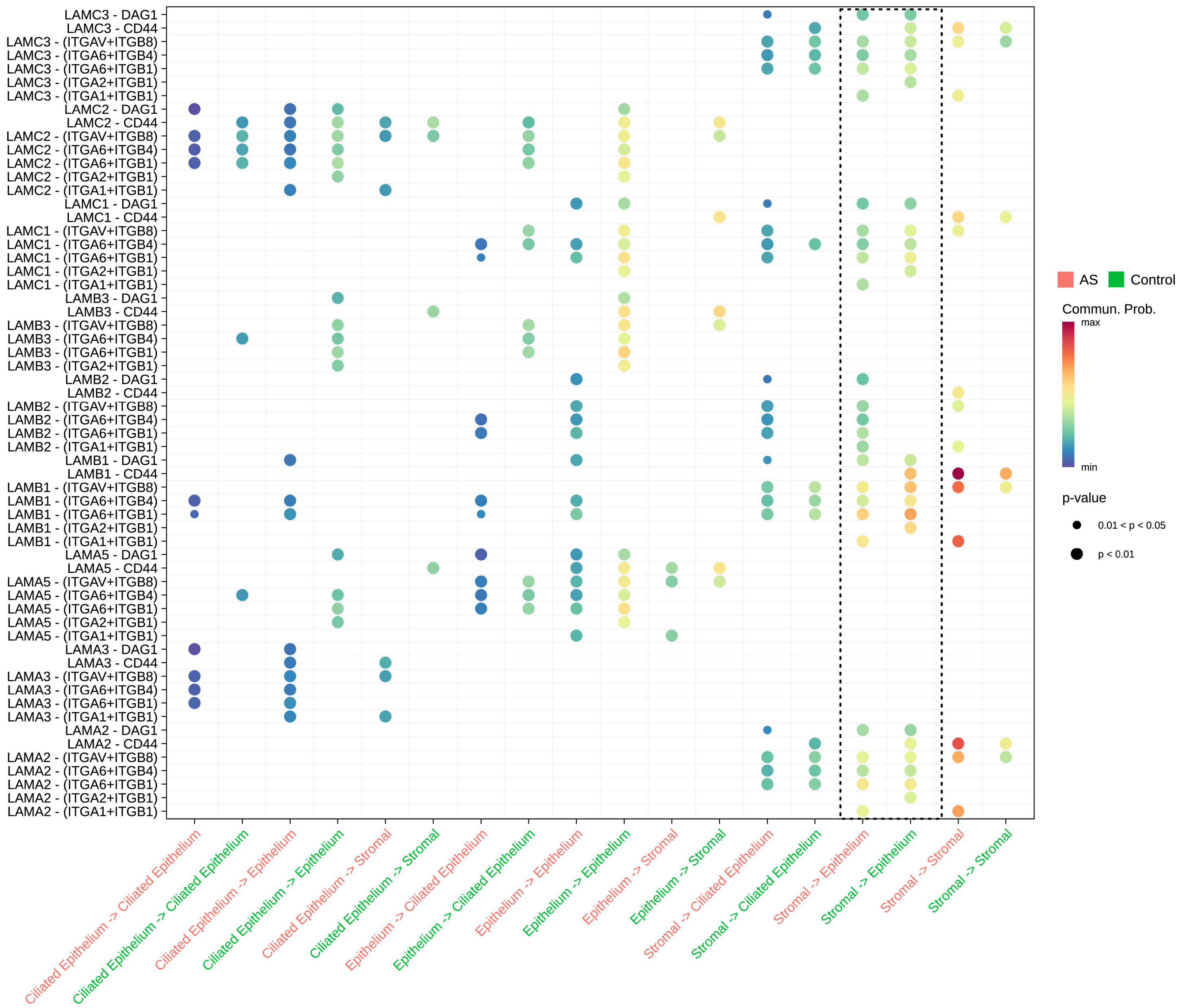

Supplementary Figure 11. Dot plot of CCC results for the LAMININ signaling pathway (two-sided Wilcoxon test). (AS in red labels, Control in green labels). Abbreviations: AS: Asherman.

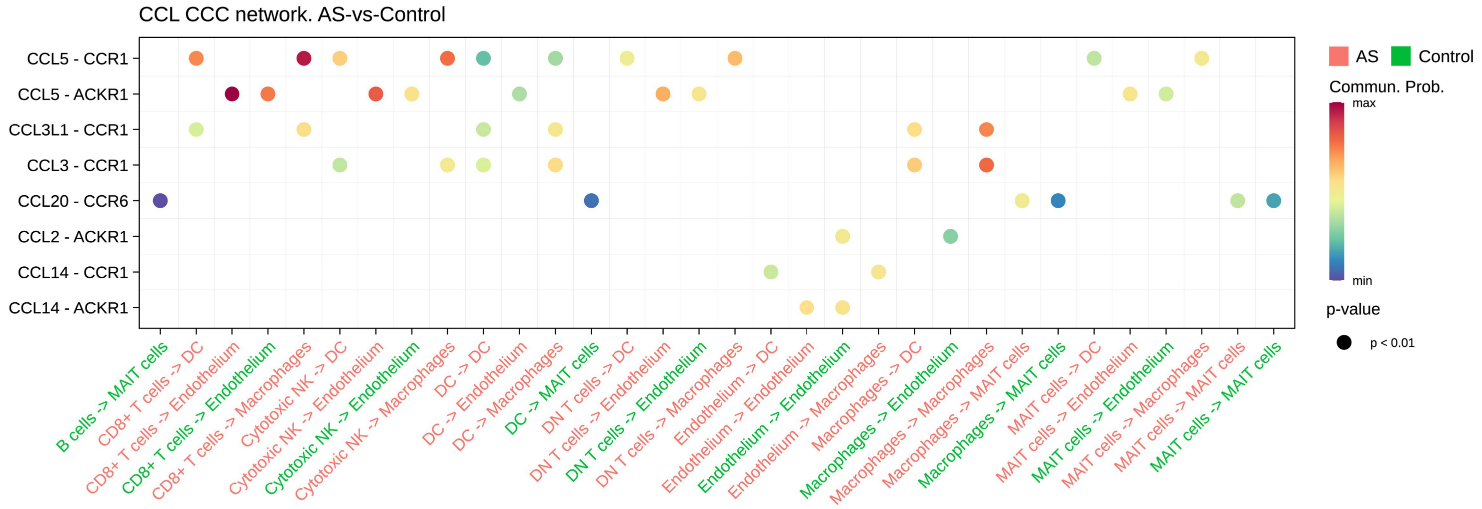

Supplementary Figure 12. Dot plot of CCC results for the CC chemokine ligand (CCL) signaling pathway (two-sided Wilcoxon test). (AS in red labels, Control in green labels). Abbreviations: AS: Asherman.

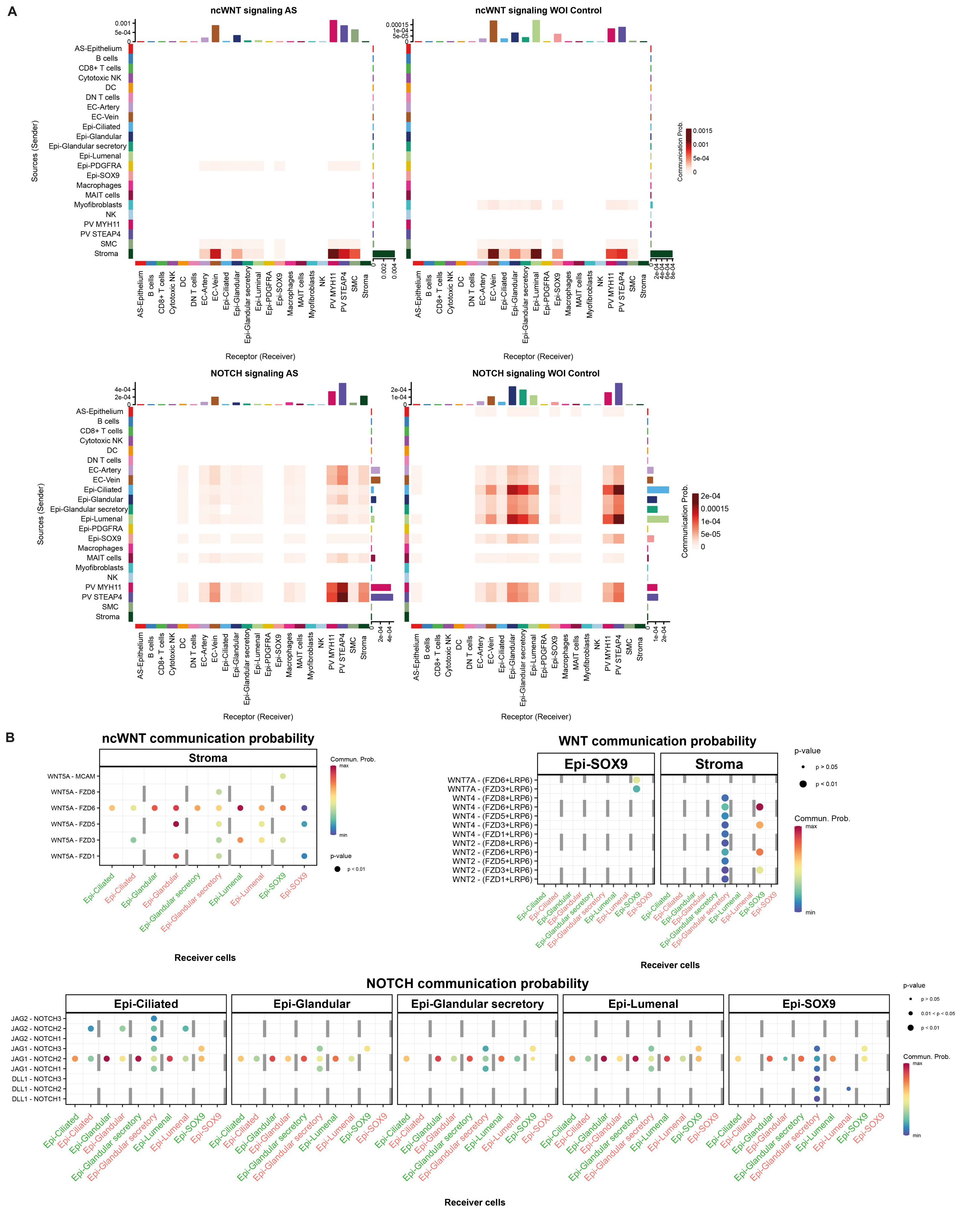

Supplementary Figure 13. CCC probabilities in pathways related to epithelial fraction differentiation. A) Heatmap displaying CCC probabilities of ncWNT and NOTCH pathways. **B)** Heatmap displaying CCC probabilities of ncWNT, WNT, and NOTCH pathways (two-sided Wilcoxon test). Abbreviations: AS: Asherman's syndrome; DC: dendritic cells; DN: double negative; EC: endothelial cell; Epi:epithelium; MAIT: mucosal-associated invariant T; NK: natural killer; PV: perivascular; SMC: smooth muscle cells.

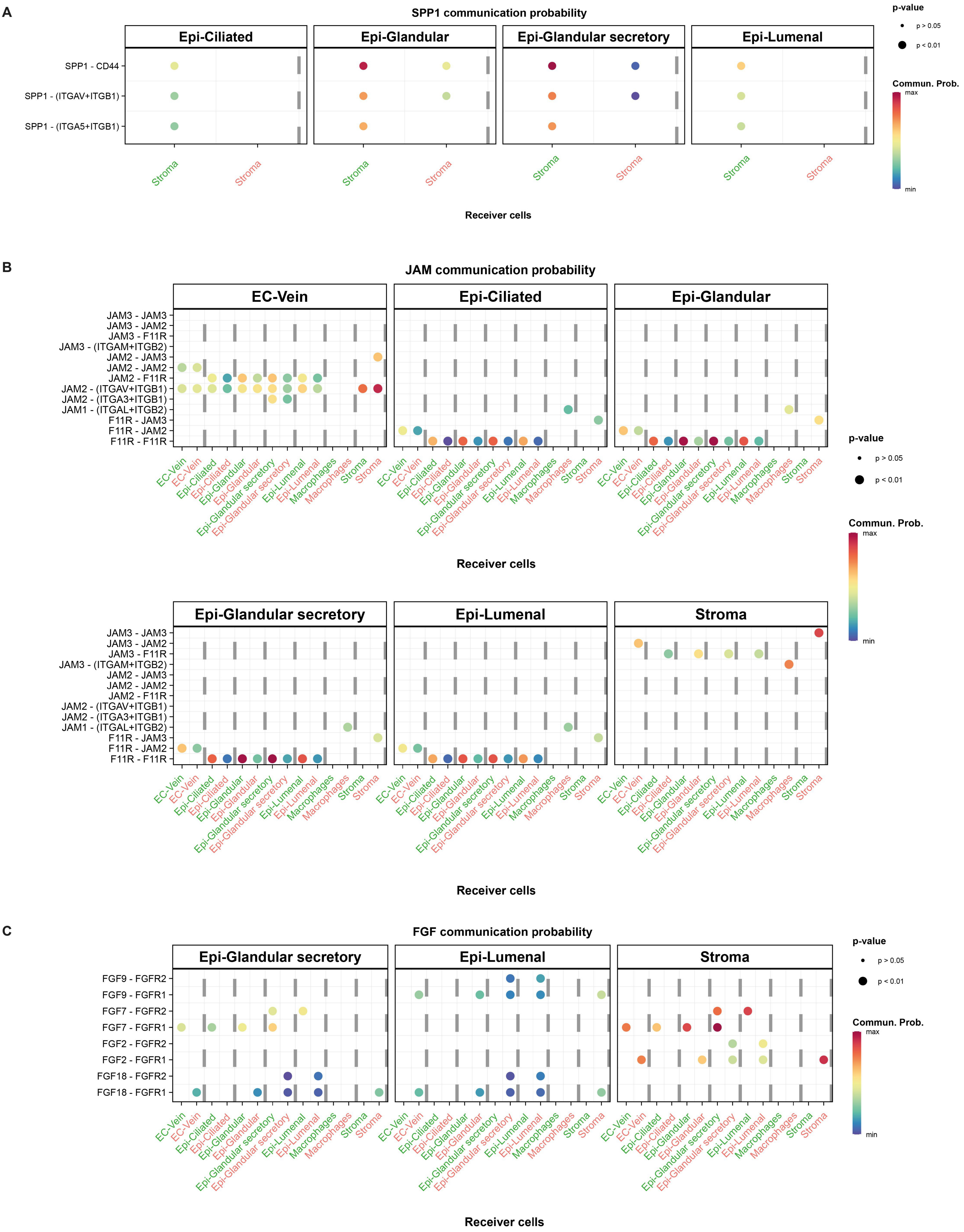

Supplementary Figure 14. CCC probabilities in pathways related to stromal decidualization, cell adhesion, and epithelial/stromal homeostasis. Dot plot displaying CCC probabilities of the **A)** SPP1 pathway (two-sided Wilcoxon test), **B)** JAM pathways (two-sided Wilcoxon test), and **C)** FGF pathways (two-sided Wilcoxon test).

Supplementary Table 1. Organoid's culture medium.

Reagent	Final concentration	Commercial brand	Reference
DMEM F12 (+Glutamine +HEPES)	1X	Thermo Fisher Scientific	31331028
Noggin	100 ng/ml	R&D Systems	6057-NG-100
RSPO-1 (R-spondin 1)	200 ng/ml	Peprotech	120-38
Insulin-Transferrin-Selenium (ITS)	1%	Life Technologies	41400045
Penicillin/streptomycin	1%	Life Technologies	15140122
N2 supplement	1%	Life Technologies	17502048
B27 supplement	2%	Life Technologies	12587010
N-acetyl L-cysteine	1.25 mM	Merck	A7250-50G
Nicotinamide	1 mM	Merck	72340-100G
A83-01	0.5 µM	Merck	SML0788-5MG
p38 inhibitor (SB202190)	10 µM	Merck	S7067
EGF (epidermal growth factor)	50 ng/ml	R&D Systems	236-EG-01M
b-FGF (basic fibroblast growth factor)	2 ng/ml	Thermo Fisher Scientific	PHG0264
FGF10 (fibroblast growth factor 10)	50 ng/ml	Peprotech	100-26
Rock Inhibitor (Y-27632)	9 µM	Merck	SCM075
