# Supplementary material for: Decoding the endometrial niche of Asherman’s Syndrome at single-cell resolution
Source: Nat Commun. 2023 Sep 21;14:5890. doi: 10.1038/s41467-023-41656-1 (PMC10514053; doi:10.1038/s41467-023-41656-1)
Supplement: Supplementary file 2 — Description of Additional Supplementary Files [file 41467_2023_41656_MOESM2_ESM.pdf]

Description of Additional Supplementary Files

Supplementary Data 1. Metadata and sample QC metrics of study subjects

Supplementary Data 2. Canonical gene markers from reference single-cell atlases to distinguish between cell types

Supplementary Data 3. Differentially expressed marker genes by cell type. Wilcoxon test two-sided was applied and p values were corrected using FDR

Supplementary Data 4. Conserved marker genes between AS and secretory-phase controls. Wilcoxon test two-side was applied and p values were corrected using FDR

Supplementary Data 5. Differentially-expressed genes between AS and secretory-phase controls. Wilcoxon test two-sided was applied and p values were corrected using FDR

Supplementary Data 6. Differentially-expressed genes between AS and WOI controls. Wilcoxon test two-sided was applied and p values were corrected using FDR

Supplementary Data 7. HTML report of differential cell-to-cell communications between AS and WOI controls

Supplementary Data 8. Differentially-expressed genes between AS, and control EEOs. Wilcoxon test two-sided was applied and p values were corrected using FDR

Supplementary Data 9. Ambient RNA genes detected using CellBender between AS and WOI control conditions

Supplementary Data 10. Cell ratios between AS and control conditions

Supplementary Data 11: sourcedata_AS_vs_control_CCC.xlsx: Contains the data related to figures 4 and supplementary figures 8, 9, 10, 11, and 12

Supplementary Data 12: sourcedata_AS_vs_WOI_control_CCC.xlsx: Contains the data related to figure 5 and supplementary figures 13 and 14
