# Supplementary material for: Decoding the endometrial niche of Asherman’s Syndrome at single-cell resolution
Source: Nat Commun. 2023 Sep 21;14:5890. doi: 10.1038/s41467-023-41656-1 (PMC10514053; doi:10.1038/s41467-023-41656-1)
Supplement: Supplementary file 9 — Supplementary Data 7 [file 41467_2023_41656_MOESM9_ESM.zip › 396088_4_data_set_8045925_s0npwb.html]

 

 

 

 
 
 


 

 Supplementary Material 

 
 
 
 
 
 
 
 
 
 
 

 

 
 


 


 

 

 


 

 


 


 


 Supplementary Material 
 2023-03-28 

 


 
 HTML Report CCCs AS vs Control WOI 
 Extended results of CCC analysis in AS vs Control WOI 
 
 Barplot of relative and absolute information flow (AS vs WOI
Control) 
 Only showing the signalling pathways that are considered significant
(p.value < 0.05) and enriched in the experimental group. This
analysis was made using the  rankNet  function and the
results of the Wilcoxon test analysis. 
   
 
 
 CCC Heatmap 
 Heatmap showing the contribution of signals to cell groups in terms
of outgoing or incoming signaling (AS vs WOI Control) 
    
 
 
 Chord plots of the detected CCC 
 Chords plots display the CCC network of the selected signaling
pathway. The lower barplot display each ligand-receptor pair
contribution of the CCC network. 
 
 Chord plots of shared signalling pathways 
 
 IL16 
   
   
 
 
 RESISTIN 
   
   
 
 
 MHC-II 
   
   
 
 
 TGFb 
   
   
 
 
 NCAM 
   
   
 
 
 EPHB 
   
   
 
 
 BMP 
   
   
 
 
 ANNEXIN 
   
   
 
 
 ITGB2 
   
   
 
 
 ICAM 
   
   
 
 
 VCAM 
   
   
 
 
 KIT 
   
   
 
 
 SELL 
   
   
 
 
 CD22 
   
   
 
 
 CD45 
   
   
 
 
 THY1 
   
   
 
 
 CLEC 
   
   
 
 
 MHC-I 
   
   
 
 
 BAFF 
   
   
 
 
 BAG 
   
   
 
 
 LIGHT 
   
   
 
 
 SEMA4 
   
   
 
 
 ANGPTL 
   
   
 
 
 PARs 
   
   
 
 
 NECTIN 
   
   
 
 
 TNF 
   
   
 
 
 SEMA3 
   
   
 
 
 PECAM1 
   
   
 
 
 CCL 
   
   
 
 
 LT 
   
   
 
 
 HSPG 
   
   
 
 
 GALECTIN 
   
   
 
 
 APP 
   
   
 
 
 ESAM 
   
   
 
 
 MPZ 
   
   
 
 
 FN1 
   
   
 
 
 LAMININ 
   
   
 
 
 COLLAGEN 
   
   
 
 
 ADGRE5 
   
   
 
 
 EDN 
   
   
 
 
 FGF 
   
   
 
 
 TENASCIN 
   
   
 
 
 JAM 
   
   
 
 
 PTPRM 
   
   
 
 
 THBS 
   
   
 
 
 CALCR 
   
   
 
 
 SPP1 
   
   
 
 
 DESMOSOME 
   
   
 
 
 TWEAK 
   
   
 
 
 CDH1 
   
   
 
 
 OCLN 
   
   
 
 
 GDF 
   
   
 
 
 EPHA 
   
   
 
 
 CDH 
   
   
 
 
 CADM 
   
   
 
 
 
 Chord plots of unique signalling pathways in AS 
 
 NRXN 
   
   
 
 
 LCK 
   
   
 
 
 ALCAM 
   
   
 
 
 CD6 
   
   
 
 
 CD226 
   
   
 
 
 SELPLG 
   
   
 
 
 TIGIT 
   
   
 
 
 PERIOSTIN 
   
   
 
 
 CD86 
   
   
 
 
 IL4 
   
   
 
 
 ICOS 
   
   
 
 
 
 Chord plots of unique signalling pathways in WOI
Control 
 
 PTH 
   
   
 
 
 NT 
   
   
 
 
 CSF3 
   
   
 
 
 VEGI 
   
   
 
 
 IL1 
   
   
 
 
 CD96 
   
   
 
 
 SEMA7 
   
   
 
 
 IL6 
   
   
 
 
 
 


 

 

 

 

 


 
 

 
 
