# Supplementary material for: Decoding the endometrial niche of Asherman’s Syndrome at single-cell resolution
Source: Nat Commun. 2023 Sep 21;14:5890. doi: 10.1038/s41467-023-41656-1 (PMC10514053; doi:10.1038/s41467-023-41656-1)
Supplement: Supplementary file 15 — Reporting Summary [file 41467_2023_41656_MOESM15_ESM.pdf]

nature portfolio

Corresponding author(s): Xavier Santamaria and Carlos Simon

Last updated by author(s): Sep 8, 2023

Reporting Summary

Nature Portfolio wishes to improve the reproducibility of the work that we publish. This form provides structure for consistency and transparency in reporting. For further information on Nature Portfolio policies, see our Editorial Policies and the Editorial Policy Checklist.

Statistics

For all statistical analyses, confirm that the following items are present in the figure legend, table legend, main text, or Methods section.

n/a	Confirmed	
☐	☒	The exact sample size (*n*) for each experimental group/condition, given as a discrete number and unit of measurement
☐	☒	A statement on whether measurements were taken from distinct samples or whether the same sample was measured repeatedly
☐	☒	The statistical test(s) used AND whether they are one- or two-sided<br>*Only common tests should be described solely by name; describe more complex techniques in the Methods section.*
☐	☒	A description of all covariates tested
☐	☒	A description of any assumptions or corrections, such as tests of normality and adjustment for multiple comparisons
☐	☒	A full description of the statistical parameters including central tendency (e.g. means) or other basic estimates (e.g. regression coefficient) AND variation (e.g. standard deviation) or associated estimates of uncertainty (e.g. confidence intervals)
☐	☒	For null hypothesis testing, the test statistic (e.g. *F*, *t*, *r*) with confidence intervals, effect sizes, degrees of freedom and *P* value noted<br>*Give P values as exact values whenever suitable.*
☒	☐	For Bayesian analysis, information on the choice of priors and Markov chain Monte Carlo settings
☐	☒	For hierarchical and complex designs, identification of the appropriate level for tests and full reporting of outcomes
☒	☐	Estimates of effect sizes (e.g. Cohen's *d*, Pearson's *r*), indicating how they were calculated

Our web collection on statistics for biologists contains articles on many of the points above.

Software and code

Policy information about availability of computer code

Data collection	Total RNA was extracted using the RNeasy Micro Kit with on-column DNase treatment (Qiagen) following the manufacturer's instructions. RNA was resuspended in 14 µl of RNase-free water, and purity and concentration determined using a NanoDrop 1000 spectrophotometer (Thermo Fisher). For cDNA synthesis, 500-1000 ng of total RNA was reverse transcribed following the manufacturer's instructions (Thermo Fisher).<br>10x-Genomics v3 libraries were prepared as per the manufacturer's instructions. Libraries were sequenced, aiming at a minimum coverage of 50,000 raw reads per cell, on an Illumina NovaSeq 6000 S2v1.5 300 cycles at a mean 40x coverage (paired-end; read 1: 26 cycles; i7 index: 8 cycles, i5 index: 0 cycles; read 2: 98 cycles)
Data analysis	Processing of scRNA-seq data was conducted with the CellRanger software suite (version 3.1.0): demultiplexing with mkfastq wrapper command of bcl2fastq (Illumina); alignment with GRCh38-3.0.0 as a reference, provided by 10x Genomics.<br>Downstream analysis for quality control, dimension reduction, clustering and visualization, were performed in RMarkdown utilizing R (4.1) scripts. The following packages and versions were used: DoubletFinder (2.0.3), scds (1.6.0) , Seurat (4.0.1), clustree (0.4.4)<br>Cell ratios differential testing was performed with edgeR (3.34.0)<br>GSEA analysis were performed with escape (1.6.0) package.<br>Cell-to-cell communication networks were constructed and analysed with CellChat (1.1.3) package.<br>Logistic regression model for organoids was developed with caret (6.0-92) and glmnet (4.1-4) packages.

For manuscripts utilizing custom algorithms or software that are central to the research but not yet described in published literature, software must be made available to editors and reviewers. We strongly encourage code deposition in a community repository (e.g. GitHub). See the Nature Portfolio guidelines for submitting code & software for further information.

Data

Policy information about availability of data

All manuscripts must include a data availability statement. This statement should provide the following information, where applicable:

- Accession codes, unique identifiers, or web links for publicly available datasets
- A description of any restrictions on data availability
- For clinical datasets or third party data, please ensure that the statement adheres to our policy

The single-cell RNAseq data generated for this manuscript has been uploaded to GEO, under accession numbers GSE215968 (https://www.ncbi.nlm.nih.gov/geo/query/acc.cgi?acc=GSE215968) and GSE216748 (https://www.ncbi.nlm.nih.gov/geo/query/acc.cgi?&acc=GSE216748). The GSE215968 dataset contains the in vivo endometrial samples from Ashermans' Syndrome patients and control endometrial samples collected under HUTER project (GA#874867) during the WOI. The GSE216748 dataset includes all the endometrial epithelial organoid samples.

Control samples of 10 healthy endometrial biopsies during the secretory phase were downloaded from GEO: GSE11197614.

Other data that support the findings of this study are available from Asherman Therapy SL, but restrictions apply to the accession of these data, which were used under license for the current clinical study, and so are not publicly available. Data are however available from the authors upon reasonable request and with permission of the Vall Hebron Ethical Committee.

Human research participants

Policy information about studies involving human research participants and Sex and Gender in Research.

Reporting on sex and gender	Since Asherman Syndrome is a uterine pathology only women have been included in the study.
Population characteristics	Requirements for participation in the study included a patient age of 18–44 years old, a BMI of 18-30, normal liver, heart, and kidney function, the absence of pregnancy, HIV, Hepatitis B or C, syphilis, or any psychiatric pathology, and a willingness to complete the study.
Recruitment	Nine patients of 34-42 years of age diagnosed with moderate and severe AS were enrolled according to the American Fertility Society (AFS) classification of IUAs 19. The diagnosis of AS was confirmed by the same surgeon (X.S.), who performed all hysteroscopies and endometrial biopsies in the secretory phase immediately before. All patients were treated with hormonal replacement therapy to synchronize cycles and treatments, and hysteroscopies were always performed during the WOI period.<br>Controls were recruited from healthy (absence of know pathologies and uterine malformations) volunteers 18-42 years-old women, with regular menstrual cycle lenght (21-35 days) and BMI between 18 and 30 Kg/m2. All samples were collected during the WOI.
Ethics oversight	The study was conducted in accordance with the International Conference on Harmonization Good Clinical Practice guidelines and the Declaration of Helsinki. All procedures involving human endometrium were approved by the Spanish Agency of Medicines and Medical Devices (Agencia Española de Medicamentos y Productos Sanitarios [AEMPS]) (April 20th, 2020) and the Clinical Research Ethics Committee at the Hospital Universitari Vall D'Hebron Barcelona, Spain (April 17th, 2020). All participants provided written informed consent.<br>For the controls, ethical approval was given by the Research Ethics Committee of the University of Tartu with approval reference 302/T-4. All participants provided written informed consent.

Note that full information on the approval of the study protocol must also be provided in the manuscript.

Field-specific reporting

Please select the one below that is the best fit for your research. If you are not sure, read the appropriate sections before making your selection.

☒ Life sciences ☐ Behavioural & social sciences ☐ Ecological, evolutionary & environmental sciences

For a reference copy of the document with all sections, see nature.com/documents/nr-reporting-summary-flat.pdf

Life sciences study design

All studies must disclose on these points even when the disclosure is negative.

Sample size	We analyzed endometrial biopsies from 9 patients of 34-42 years of age affected with moderate and severe AS. A total of 40,336 AS cells were assessed.<br>For comparison against Asherman condition we used two control populations:<br>a) controls from GSE111976 (PMID: 32929266) of (n=10) healthy endometrium biopsies, collected during the secretory phase of the menstrual cycle, were integrated with Asherman samples. 69,202 cells were analyzed from this dataset in this study.<br>b) Endometrial biopsies of control individuals were collected from fertile and asymptomatic women during the WOI (n=6).

Data exclusions	Following pre-stablished criteria, low-quality cells in samples were filtered out based on 3 quality control metrics: (i) low detected number of genes (ii) low detected number of counts, or (iii) high mitochondrial percentage of read counts. Cell-barcodes suspicious of containing more than one single cell were also removed.
Replication	Each conclusion presented in the study is supported by no less than 5 biological replicates (women) and high statistical significance.
Randomization	No Randomization has ben performed
Blinding	No blinding has been performed

Reporting for specific materials, systems and methods

We require information from authors about some types of materials, experimental systems and methods used in many studies. Here, indicate whether each material, system or method listed is relevant to your study. If you are not sure if a list item applies to your research, read the appropriate section before selecting a response.

Materials & experimental systems

n/a	Involved in the study
☒	☐ Antibodies
☒	☐ Eukaryotic cell lines
☒	☐ Palaeontology and archaeology
☒	☐ Animals and other organisms
☒	☐ Clinical data
☒	☐ Dual use research of concern

Methods

n/a	Involved in the study
☒	☐ ChIP-seq
☒	☐ Flow cytometry
☒	☐ MRI-based neuroimaging
